# KLRG1^+^CD28^⁻^CD57^+^ Ag85A-specific CD8^+^ T cells mark a severity-associated terminal-differentiation imprint in pulmonary tuberculosis

**DOI:** 10.3389/fcimb.2026.1938907

**Published:** 2026-10-01

**Authors:** Jiaming Wu, Sufang Chen, Rui Qiang, Longji Cheng, Minyan Yao, Zhen Zhang, Xin Yu, Junchi Xu, Jianping Zhang

**Affiliations:** 1 Suzhou Medical College of Soochow University, Suzhou, China; 2 Department of Clinical Laboratory, The Fifth People’s Hospital of Suzhou & The Affiliated Infectious Diseases Hospital of Soochow University, Suzhou, China; 3 Department of Clinical Laboratory, The Second Affiliated Hospital of Soochow University, Suzhou, China; 4 Department of Tuberculosis, The Fifth People’s Hospital of Suzhou & The Affiliated Infectious Diseases Hospital of Soochow University, Suzhou, China

**Keywords:** Ag85A-specific CD8^+^ T cells, disease severity, host–pathogen interaction, immune correlate, KLRG1^+^CD28⁻CD57^+^ phenotype, Mycobacterium tuberculosis, pulmonary tuberculosis, terminal differentiation

## Abstract

**Background:**

Pulmonary tuberculosis (PTB) is characterized by prolonged host–pathogen interaction, chronic granulomatous inflammation, and sustained exposure to Mycobacterium tuberculosis (Mtb) antigens. How Mtb antigen-specific CD8^+^ T cell differentiation states relate to clinical disease activity and severity remains incompletely defined. This study investigated the clinical relevance of a terminal differentiation-associated phenotype within Ag85A-specific CD8^+^ T cells across clinically defined states of Mtb infection and disease.

**Methods:**

A total of 124 participants were enrolled, including 55 patients with PTB, 33 individuals with latent tuberculosis infection (LTBI), and 36 healthy controls (HCs). Peripheral blood mononuclear cells were analyzed by flow cytometry to quantify KLRG1^+^CD28^⁻^CD57^+^ cells among HLA-A*02:01-restricted Ag85A_48_-_56_ tetramer^+^CD8^+^ T cells. Associations with active disease, sputum smear status, clinical severity, selected host-status parameters, and exploratory treatment-related outcomes were assessed.

**Results:**

Patients with PTB showed a significantly higher proportion of KLRG1^+^CD28^⁻^CD57^+^ Ag85A-specific CD8^+^ T cells than HCs. This phenotype was enriched in active PTB and was associated with clinically relevant features of disease burden, including sputum smear positivity, higher smear grade, lymphocyte imbalance, and severe pulmonary involvement. Patients with severe PTB had higher proportions of this subset than patients with non-severe PTB. In receiver operating characteristic analysis, this phenotype showed exploratory in-cohort discriminative potential for distinguishing severe from non-severe PTB, with an apparent area under the curve of 0.8333 (95% CI, 0.7268–0.9398; P<0.0001); patient-level bootstrap resampling yielded a bias-corrected AUC of 0.8333.

**Conclusions:**

The accumulation of KLRG1^+^CD28^⁻^CD57^+^ Ag85A-specific CD8^+^ T cells is closely associated with active and clinically severe PTB. Rather than establishing a causal mechanism, these findings identify an antigen-specific, terminal differentiation-associated CD8^+^ T-cell phenotype as a potential translational immune correlate of disease severity in human Mtb infection. Its discriminative ability should be regarded as preliminary and requires external validation in larger, prospective, and independently recruited cohorts.

## 1 Introduction

Tuberculosis remains a major infectious disease whose occurrence and clinical course are shaped by a prolonged and dynamic interaction between Mycobacterium tuberculosis (Mtb) and the host immune system (Kaufmann and Dorhoi, 2013; Chandra et al., 2022; Trajman et al., 2025). Unlike many acute bacterial pathogens, Mtb can establish long-term infection, persist within complex granulomatous lesions, evade or withstand immune-mediated clearance, and maintain antigenic stimulation over extended periods (Ramakrishnan, 2012; Kaufmann and Dorhoi, 2013; Chandra et al., 2022; Trajman et al., 2025). This chronic host–pathogen relationship creates a distinctive immunological environment in which protective immunity, bacterial containment, inflammatory pathology, and immune dysfunction coexist (Ramakrishnan, 2012; Kaufmann and Dorhoi, 2013; Chandra et al., 2022; Trajman et al., 2025). Understanding how Mtb-specific T-cell populations are shaped during different clinical states of infection is therefore essential for clarifying the immune features associated with active disease and disease severity.

Cell-mediated immunity is central to the control of Mtb infection (Behar, 2013; Lin and Flynn, 2015; Trajman et al., 2025). CD4^+^ T cells have traditionally received the greatest attention in tuberculosis immunology; however, CD8^+^ T cells also contribute to host defense through cytokine production, recognition of infected cells, and cytotoxic effector functions (Behar, 2013; Lin and Flynn, 2015; Yang et al., 2018). During persistent infection, antigen-specific CD8^+^ T cells may undergo progressive differentiation characterized by altered proliferative capacity, changes in co-stimulatory and inhibitory receptor expression, and accumulation of highly differentiated effector populations (Rozot et al., 2013; Goronzy and Weyand, 2019; Liu et al., 2023; Terekhova et al., 2025). These changes are particularly relevant in tuberculosis, where prolonged antigen exposure and sustained inflammatory activation may accompany bacterial persistence, tissue inflammation, and high bacillary burden, and clinical disease activity (Kaufmann and Dorhoi, 2013; Chandra et al., 2022; Chang et al., 2025; Trajman et al., 2025). Nevertheless, the clinical significance of highly differentiated or terminal differentiation-associated CD8^+^ T cell phenotypes in human Mtb infection remains insufficiently characterized.

Ag85A is a major secreted Mtb antigen belonging to the antigen 85 complex, a family of mycolyl-transferases involved in mycobacterial cell-wall biology and widely studied in Mtb-specific T-cell immunity (Huygen, 2014). Ag85A-specific CD8^+^ T cells represent a useful antigen-specific compartment for assessing cellular immune differentiation during Mtb infection, and HLA-A*02:01-restricted Ag85A epitopes, including Ag85A_48_-_56_, have been reported to elicit human CD8^+^ cytotoxic T-cell responses (Smith et al., 2000). Previous clinical studies have shown that Mtb-specific CD8^+^ T cells differ between individuals with latent tuberculosis infection and patients with active tuberculosis in phenotypic and functional features (Rozot et al., 2013). However, most studies have focused on the magnitude of antigen-specific responses or cytokine-producing T-cell subsets, whereas less is known about whether the differentiation status of Ag85A-specific CD8^+^ T cells reflects distinct clinical states such as latent tuberculosis infection, active pulmonary tuberculosis, smear-positive disease, or severe pulmonary involvement (Rozot et al., 2013; Yu et al., 2022; Yu et al., 2024). This gap is important because antigen-specific T-cell phenotypes may provide information beyond conventional measures of immune activation and may help identify host immune correlates of Mtb burden and disease severity.

Immunosenescence in T cells refers to a state of altered immune differentiation and function that may arise with aging, chronic antigen exposure, or sustained inflammatory stimulation (Nikolich-Žugich, 2018; Goronzy and Weyand, 2019; Liu et al., 2023; Terekhova et al., 2025). In contrast to classical cellular senescence in non-immune cells, which is often characterized by DNA damage responses, irreversible cell-cycle arrest, and senescence-associated secretory programs, T-cell immunosenescence is commonly assessed by phenotypic and functional changes such as loss of co-stimulatory receptors, reduced proliferative capacity, and accumulation of terminally differentiated populations (Nikolich-Žugich, 2018; Gorgoulis et al., 2019; Goronzy and Weyand, 2019; Rodriguez et al., 2020; Liu et al., 2023; Terekhova et al., 2025). Among these markers, CD28, KLRG1, and CD57 are frequently used to characterize highly differentiated T-cell states (Brenchley et al., 2003; Henson and Akbar, 2009; Rodriguez et al., 2020; Liu et al., 2023; Terekhova et al., 2025). CD28 is a key co-stimulatory receptor required for optimal T-cell activation, IL-2 production, and proliferative renewal; loss of CD28 is generally associated with reduced proliferative potential and advanced differentiation (Goronzy and Weyand, 2019; Huff et al., 2019; Yu et al., 2022; Liu et al., 2023; Terekhova et al., 2025). KLRG1 is an inhibitory receptor linked to terminal effector differentiation, whereas CD57 marks a highly differentiated T-cell population with limited replicative capacity (Brenchley et al., 2003; Henson and Akbar, 2009; Hu et al., 2018). The combined KLRG1^+^CD28^⁻^CD57^+^ phenotype was therefore used operationally to identify a highly differentiated, terminal differentiation-associated CD8^+^ T cell population. Although these markers have also been linked to immunosenescence in previous studies, they do not by themselves establish irreversible T-cell senescence.

Although senescence-associated immune features have been described in tuberculosis-related inflammatory settings, their relevance should be interpreted within the specific biology of Mtb infection rather than extrapolated directly from other chronic infections (Bobak et al., 2022; Chandra et al., 2022; Sun et al., 2025). Mtb persistence is not merely a background source of inflammation; it is a defining feature of the pathogen’s survival strategy (Ramakrishnan, 2012; Chandra et al., 2022). Granulomatous inflammation, repeated or sustained antigen recognition, and ongoing immune activation may together influence the differentiation trajectory of Mtb-specific T cells (Ramakrishnan, 2012; Kaufmann and Dorhoi, 2013; Rozot et al., 2013; Chandra et al., 2022; Trajman et al., 2025). However, whether an immunosenescence-associated phenotype within Ag85A-specific CD8^+^ T cells is associated with active disease, smear-defined mycobacterial burden, radiographic or clinical severity, and treatment-related outcomes in human PTB remains under-characterized, particularly in observational clinical cohorts.

In the present study, we analyzed KLRG1, CD28, and CD57 expression within Ag85A_48_-_56_ tetramer^+^CD8^+^ T cells from patients with active PTB, individuals with LTBI, and healthy controls. We hypothesized that active PTB is accompanied not only by quantitative changes in Ag85A-specific CD8^+^ T cells, but also by a clinically meaningful terminal-differentiation imprint within this antigen-specific compartment. Specifically, we investigated whether KLRG1^+^CD28^⁻^CD57^+^ Ag85A-specific CD8^+^ T cells map onto a clinical continuum encompassing latent infection, active disease, smear-defined bacillary burden, selected host-status parameters, and severe pulmonary involvement. By focusing on an antigen-specific CD8^+^ T-cell population, this study provides a translational observational assessment of how Mtb-associated immune differentiation relates to disease phenotype, while recognizing that causal mechanisms require further validation through functional, longitudinal, and independent cohort studies.

## 2 Materials and methods

### 2.1 Study subjects

This was a single-center observational study comprising a cross-sectional comparison among patients with pulmonary tuberculosis (PTB), individuals with latent tuberculosis infection (LTBI), and healthy controls (HCs), together with an exploratory longitudinal follow-up of sputum smear-positive patients with PTB. Immunophenotypic measurements were performed at baseline, and selected treatment-related outcomes were subsequently assessed during anti-tuberculosis therapy (**Figure 1**).

**Figure 1:**
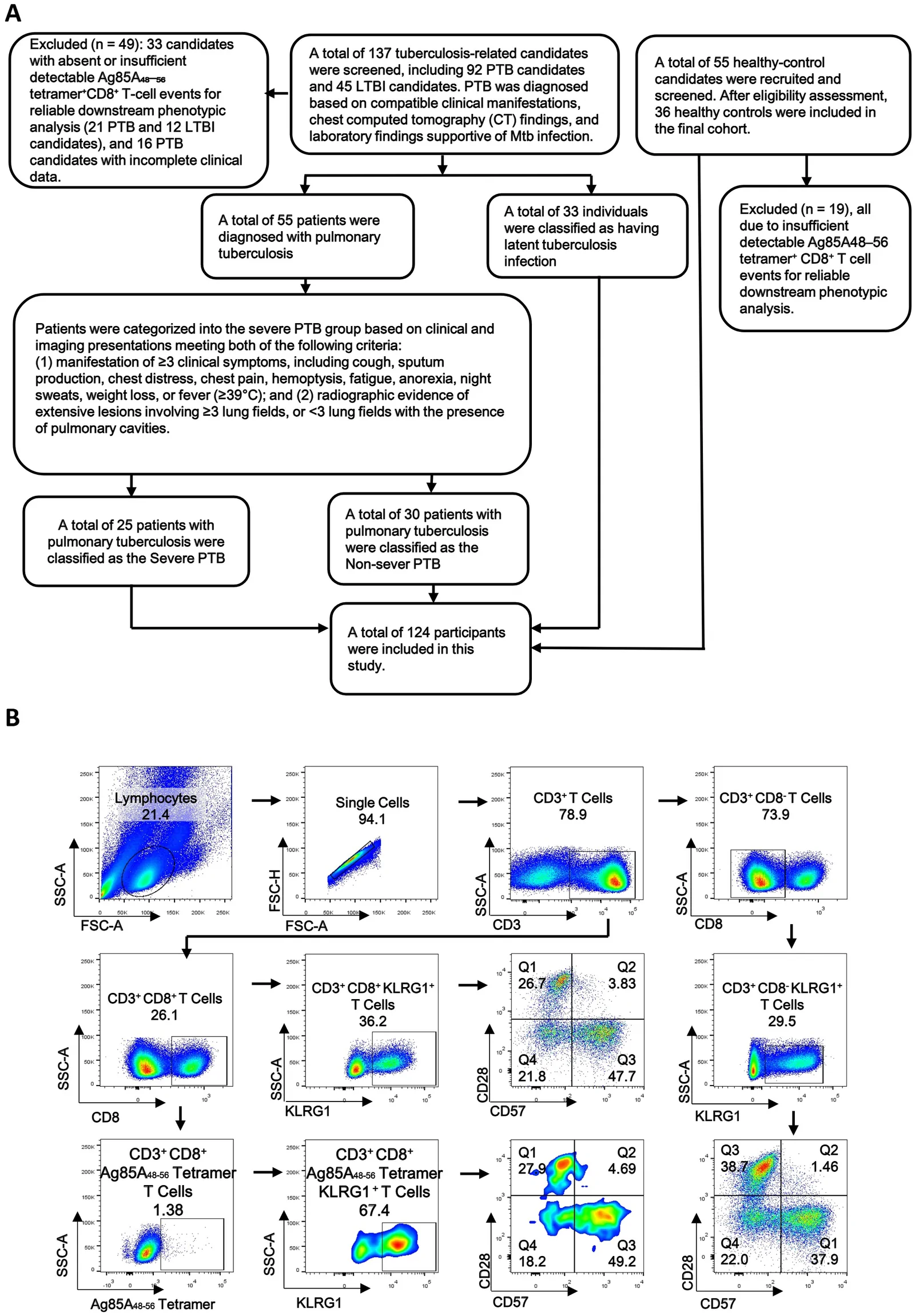
Study enrollment, clinical classification, and flow-cytometric gating strategy. **(A)** Flow diagram showing participant screening, exclusion, enrollment, and clinical classification. A total of 192 candidates were initially screened, including 92 PTB candidates, 45 LTBI candidates, and 55 healthy-control candidates. Fifty-two candidates were excluded because of absent or insufficient detectable Ag85A_48_-_56_ tetramer^+^CD8^+^ T cell events for reliable downstream phenotypic analysis, including 21 PTB candidates, 12 LTBI candidates, and 19 healthy-control candidates. An additional 16 PTB candidates were excluded because of incomplete clinical data. After these exclusions, 124 participants were included in the final analytic cohort, comprising 55 patients with pulmonary tuberculosis (PTB), 33 individuals with latent tuberculosis infection (LTBI), and 36 healthy controls (HCs). Among the 55 patients with PTB, 25 were classified as having severe PTB and 30 as having non-severe PTB according to the predefined clinical and radiological criteria. **(B)** Representative flow-cytometric gating strategy used to identify T-cell subsets and Ag85A-specific CD8^+^ T cells. Lymphocytes were first gated according to forward scatter and side scatter, followed by single-cell gating and identification of CD3^+^ T cells. CD3^+^ T cells were further separated into CD3^+^CD8^+^ and CD3^+^CD8^⁻^ T cell subsets. KLRG1^+^ cells were then identified within the indicated T-cell populations, and CD28 and CD57 expression was analyzed to define the KLRG1^+^CD28^⁻^CD57^+^ phenotype. Ag85A-specific CD8^+^ T cells were identified as HLA-A*02:01-restricted Ag85A_48_-_56_ tetramer^+^ cells within the CD3^+^CD8^+^ T cell gate, followed by analysis of KLRG1, CD28, and CD57 expression within the tetramer-positive population. Numbers shown in the plots indicate the percentage of cells within the corresponding parent gate. An Ag85A_48_-_56_ tetramer FMO control and a marker-specific KLRG1 FMO control were used to assist definition of the tetramer-positive and KLRG1-positive gates, respectively. A CD28/CD57 double-minus control was used as a background reference for downstream quadrant placement. Absolute tetramer-positive event counts were retrospectively reviewed for quality-control assessment. All samples included in the final analytic cohort contained ≥100 Ag85A_48_-_56_ tetramer^+^CD8^+^ T cell events and ≥50 tetramer^+^CD8^+^KLRG1^+^ events for downstream CD28/CD57 phenotyping. The representative plots shown in panel B were generated from a sample included in the final analytic cohort and illustrate the revised gating strategy used for the downstream analyses.

From June 2025 to April 2026, a total of 192 candidates were initially screened at The Fifth People’s Hospital of Suzhou & The Affiliated Infectious Diseases Hospital of Soochow University, including 92 PTB candidates, 45 individuals with LTBI, and 55 healthy-control candidates. During participant selection, 52 candidates were excluded because of absent or insufficient detectable Ag85A_48_-_56_ tetramer^+^CD8^+^ T cell events for reliable downstream phenotypic analysis, including 21 PTB candidates, 12 LTBI candidates, and 19 healthy-control candidates. An additional 16 PTB candidates were excluded because of incomplete clinical data. After these exclusions, the final study cohort comprised 124 participants, including 55 patients with PTB, 33 individuals with LTBI, and 36 healthy controls (HCs) (**Figure 1A**). For age-stratified analyses, participants were classified consistently into three predefined age groups: Group 1, ≤12 years; Group 2, 13–39 years; and Group 3, ≥40 years.

HCs were required to have no known underlying comorbidities, including hypertension, diabetes mellitus, chronic obstructive pulmonary disease, and no clinical symptoms suggestive of tuberculosis or respiratory illness, including cough, expectoration, hemoptysis, chest pain, dyspnea, fatigue, anorexia, weight loss, fever, or night sweats. HCs also had no known history of close TB contact, no previous diagnosis of pulmonary or extrapulmonary TB, including bronchial TB, tuberculous pleurisy, or tuberculous polyserositis, normal chest radiographs, and negative interferon-γ release assay (IGRA) results.

Individuals were classified as having LTBI if they had a positive IGRA result in the absence of clinical or radiographic evidence of active TB. Specifically, LTBI participants had no symptoms suggestive of active pulmonary or extrapulmonary TB, including cough, expectoration, hemoptysis, chest pain, dyspnea, fatigue, anorexia, weight loss, fever, or night sweats, and had normal chest radiographs. Individuals with a previous diagnosis of active TB, a history of anti-tuberculosis treatment, or underlying comorbidities such as hypertension, diabetes mellitus, chronic obstructive pulmonary disease were excluded from the LTBI cohort.

PTB was diagnosed at the study hospital based on compatible clinical manifestations, chest computed tomography (CT) findings, and laboratory findings supportive of Mtb infection according to routine institutional diagnostic procedures. Laboratory assessment included sputum acid-fast bacilli smear microscopy, supportive immunological testing, and, where available in routine clinical records, nucleic acid amplification testing, including Xpert MTB/RIF or TB-DNA assays. These nucleic acid amplification results were available only for a subset of patients and were not performed uniformly across the entire PTB cohort. Immunological testing was interpreted in conjunction with clinical, radiological, and microbiological findings and was not considered sufficient alone to establish active PTB.

For smear-negative patients or patients without available nucleic acid amplification results, the diagnosis of PTB was established by treating tuberculosis specialists based on the overall clinical, radiological, and laboratory assessment. Therefore, PTB classification in this study reflected routine institutional clinical diagnosis rather than uniform microbiological confirmation by culture or nucleic acid testing in all cases.

PTB patients were further classified as severe or non-severe according to predefined clinical and radiological criteria. Severe PTB was defined by the presence of both of the following: (1) at least three symptoms among cough, sputum production, chest tightness, chest pain, hemoptysis, fatigue, loss of appetite, night sweats, weight loss, and body temperature ≥39 °C; and (2) extensive pulmonary involvement affecting ≥3 lung fields, or involvement of <3 lung fields accompanied by pulmonary cavitation. Patients who did not meet these combined criteria were classified as having non-severe PTB.

Because the primary severe-versus-non-severe classification used in this study was based on study-specific clinical and radiological criteria, disease severity was additionally benchmarked using complementary published scoring approaches. Clinical severity was retrospectively assessed in all 55 patients with PTB using the standard Bandim TB score according to the published scoring algorithm (Wejse et al., 2008). All required clinical and anthropometric variables, including body mass index (BMI) and mid-upper arm circumference (MUAC), were available for calculation of the score. A TB score ≥8 was used to define severe clinical disease, whereas a TB score <8 was classified as non-severe for sensitivity analysis (Virenfeldt et al., 2014).

Radiological disease burden was additionally quantified using the CT-based weighted profusion scoring method described by Chu et al. (2019). Briefly, both lungs were divided into six zones, and the total weighted profusion score was calculated as the summed profusion score ×100/24, with an additional 40 points assigned when pulmonary cavitation was present, according to the published scoring method (Chu et al., 2019). CT score was available for 52 of the 55 patients with PTB. In the present study, CT score was primarily analyzed as a continuous measure of radiological disease burden. The original study-specific severe-versus-non-severe classification remained the primary severity definition, whereas TB score- and CT score-based analyses were performed as complementary sensitivity analyses.

Because Bacillus Calmette-Guérin(BCG) vaccination at birth is routinely implemented in China under the national immunization program, prior BCG exposure was expected to be common in the local population. Therefore, baseline Ag85A-related immune memory in HCs was considered an expected background condition rather than an exclusion criterion.

The Ag85A_48_-_56_ tetramer used in this study is HLA-A*02:01-restricted; however, formal HLA genotyping was not performed. Tetramer-based downstream analyses were therefore restricted to samples in which a discernible Ag85A_48_-_56_ tetramer^+^CD8^+^ T cell population could be identified after sequential gating. In total, 52 candidates were excluded because of absent or insufficient detectable tetramer-positive events for reliable downstream phenotypic analysis, including 21 PTB candidates, 12 LTBI candidates, and 19 healthy-control candidates. Retrospective review showed that these excluded samples contained a median of 8 tetramer-positive events (IQR, 4-10.25; range, 0-28). Because formal HLA-A*02:01 genotyping was not performed, absent or very low tetramer staining could not be definitively attributed to HLA-A*02:01 mismatch, a very low frequency of Ag85A-specific CD8^+^ T cells, or technical factors. Therefore, these excluded samples were not interpreted as evidence of HLA-A*02:01 negativity. Because a numerical minimum tetramer-positive event threshold had not been prospectively documented, absolute Ag85A_48_-_56_ tetramer^+^CD8^+^ T cell event counts were retrospectively reviewed as a quality-control measure. Following application of the revised gating strategy, all 124 samples in the final analytic cohort retained ≥100 tetramer^+^CD8^+^ T cell events; therefore, no additional samples were excluded.

Participants were excluded if they met any of the following criteria: (1) concomitant malignancy, immune system disorders, or severe hepatic or renal dysfunction; (2) receipt of systemic immunosuppressive therapy within the preceding 3 months; (3) incomplete clinical or laboratory data; or (4) insufficient detectable Ag85A_48_-_56_ tetramer^+^CD8^+^ T cell events for reliable downstream phenotypic analysis.

### 2.2 Ethics statement

Written informed consent was obtained from all adult participants before enrollment. For participants younger than 18 years of age, written informed consent was obtained from a parent or legal guardian, and assent was obtained from the participant when appropriate.

### 2.3 Clinical laboratory assessments

Peripheral venous blood samples were collected at baseline before or at the time of clinical evaluation for routine hematological, biochemical, and inflammatory cytokine assessments. All clinical laboratory measurements used in this study were obtained from baseline clinical laboratory data generated by the study hospital.

For complete blood counts, venous blood was collected into EDTA anticoagulant tubes, gently mixed, and analyzed using an automated hematology analyzer according to routine clinical laboratory procedures. Hematological variables used in the present study included leukocyte count, neutrophil percentage, lymphocyte percentage, and other routinely reported blood cell parameters.

For biochemical measurements, venous blood was collected into serum-separator tubes containing clot activator or into heparin anticoagulant tubes, according to the requirements of the clinical laboratory assays. After centrifugation, serum or plasma was separated and analyzed using an automated biochemical analyzer according to standard laboratory procedures. Biochemical variables used in the present study included albumin and other routinely reported biochemical indices.

For inflammatory cytokine assessment, venous blood was collected concurrently at baseline, and plasma was isolated after centrifugation. Twelve plasma cytokines were measured as part of the routine clinical cytokine testing panel at the study hospital, including IL-5, IFN-α, IL-2, IL-6, IL-1β, IL-10, IFN-γ, IL-8, IL-17, IL-4, IL-12p70, and TNF-α. These cytokine measurements were performed by the clinical laboratory according to the hospital’s standard operating procedures and the manufacturers’ instructions for the clinical testing platform in use. Plasma cytokine measurements were analyzed separately from intracellular cytokine staining assays performed in PBMC stimulation experiments.

Sputum specimens were obtained by early-morning deep expectoration, placed in sterile screw-cap containers, and promptly transported to the clinical laboratory. Smears were prepared according to standard procedures, stained for acid-fast bacilli, and examined microscopically by trained laboratory personnel. Sputum smear results were recorded as negative or positive, and positive smears were graded as 1+, 2+, or 3+ according to routine clinical laboratory reporting criteria.

### 2.4 GEO dataset selection and acquisition (GSE158769)

The public transcriptomic dataset GSE158769 was obtained from the NCBI Gene Expression Omnibus (GEO) repository. The dataset was downloaded and used for secondary analysis to provide complementary transcriptomic context related to immune activation and senescence-associated signatures in TB. This analysis involved only publicly available de-identified data and did not involve new sample collection or additional participant recruitment.

### 2.5 Clinical follow-up and outcome definitions

Sputum smear-positive patients with PTB were followed during anti-tuberculosis treatment using routine clinical records and follow-up sputum smear results. The follow-up analysis was exploratory and descriptive and was performed to compare baseline proportions of KLRG1^+^CD28^⁻^CD57^+^ cells among Ag85A-specific CD8^+^ T cells across small patient groups defined by subsequent follow-up status. Exploratory groups included patients who achieved sputum smear conversion after 2 months of treatment, patients who remained smear-positive after 6 months of treatment, and patients who died during follow-up. Two-month smear conversion was defined as conversion from a positive baseline sputum smear to a negative sputum smear after 2 months of anti-tuberculosis treatment. Persistent smear positivity was defined as failure to achieve sputum smear conversion after 6 months of treatment. Death was recorded as an all-cause event without assuming tuberculosis-specific causality. These categories were used solely for preliminary descriptive comparison and were not intended as formal treatment-outcome definitions, prognostic endpoints, or evidence for prediction of treatment outcome.

### 2.6 Flow cytometry

Peripheral blood samples of 3–5 mL were collected from PTB patients, LTBI individuals, and healthy controls using lithium heparin as the anticoagulant. All samples were processed within 12 h of collection. Blood samples were centrifuged at 500 × g for 5 min to separate plasma, and the plasma supernatant was removed, and the remaining cellular fraction was used for PBMC isolation. The remaining cellular fraction was diluted 1:1 with phosphate-buffered saline (PBS). Peripheral blood mononuclear cells (PBMCs) were isolated using human peripheral blood lymphocyte separation medium (TBD; Cat. No. LTS1077; Tianjin, China). Residual red blood cells were lysed using RBC lysis buffer (BD Biosciences; Cat. No. 555899).

For tetramer and surface-marker staining, 10μL of Clear Back reagent (MBL; Cat. No. MTG-001) was added to 50μL of cell suspension and incubated at room temperature for 5 min to reduce nonspecific staining. Cells were then stained with the HLA-A*02:01-restricted Ag85A_48_-_56_ T-Select MHC tetramer (MBL; Cat. No. TS-M128-2) at 4 °C for 45 min in the dark. After washing with PBS, surface staining was performed using fluorochrome-conjugated antibodies against CD3 (BioLegend; Cat. No. 317346), CD8 (BioLegend; Cat. No. 344707), CD28 (BioLegend; Cat. No. 377611), KLRG1 (BioLegend; Cat. No. 367717), and CD57 (BioLegend; Cat. Nos. 359611 and 359623) at 4 °C for 25 min in the dark. Cells were then washed twice with PBS and resuspended in 500μL of 1× PBS for acquisition.

For intracellular cytokine staining after the revised *in vitro* stimulation experiments, cytokine-stimulation and protein-transport-inhibition conditions were performed as described in Section 2.7. Briefly, brefeldin A and monensin were applied during the final 4 h of culture to all experimental conditions, whereas PMA/ionomycin was restricted to the separate positive-control condition. Following culture, cells were harvested and stained with the HLA-A*02:01-restricted Ag85A_48_-_56_ tetramer and the indicated surface antibodies. Cells were then fixed and permeabilized using the BD Cytofix/Cytoperm™ Fixation/Permeabilization Kit (BD Biosciences; Cat. No. 554714). After washing with BD Perm/Wash™ Buffer, cells were stained intracellularly with antibodies against IFN-γ (BioLegend; Cat. No. 506517) and TNF-α (BioLegend; Cat. No. 502929). Cells were washed, resuspended in PBS, and acquired on a BD FACSAria™ III Cell Sorter.

Flow cytometric data were analyzed using FlowJo software version 10.8. Lymphocytes were first identified using a broad FSC-A/SSC-A gate encompassing the lymphocyte distribution while excluding obvious debris, followed by singlet gating and identification of CD3^+^CD8^+^ T cells. Ag85A_48_-_56_ tetramer-positive events were defined using an Ag85A_48_-_56_ tetramer fluorescence-minus-one (FMO) control. To minimize inclusion of borderline tetramer-negative events, the tetramer positive gate was restricted to the clearly separated sparse tetramer-positive population beyond the FMO-defined background region. KLRG1 positive cells were subsequently identified within Ag85A_48_-_56_ tetramer^+^CD8^+^ T cells using a marker-specific KLRG1 FMO control. For downstream CD28/CD57 quadrant analysis, a CD28/CD57 double-minus control was used as the background reference for quadrant placement. The KLRG1^+^CD28^⁻^CD57^+^ subset was quantified within the Ag85A_48_-_56_ tetramer^+^CD8^+^ T cell population. The revised gating strategy was applied consistently across all samples and downstream analyses.

Following application of the revised gating strategy, absolute Ag85A_48_-_56_ tetramer^+^CD8^+^ T cell event counts were re-evaluated in all samples included in the final analytic cohort. All 124 included samples retained ≥100 tetramer^+^CD8^+^ T cell events. A conservative threshold of ≥100 tetramer^+^CD8^+^ events was therefore retained as a retrospective quality-control and sensitivity criterion, and no additional samples were excluded.

Before the formal experiments, a paired pilot analysis was performed to assess whether inclusion of a viability dye affected identification of Ag85A_48_-_56_ tetramer^+^CD8^+^ T cells or subsequent KLRG1/CD28/CD57 phenotyping. In separate control experiments, an irrelevant HIV peptide–MHC tetramer was used to assess nonspecific tetramer staining. A formally confirmed HLA-mismatched control was not included because prospective HLA-A*02:01 genotyping was not performed. Because CD28/CD57 quadrant analysis was performed downstream of the KLRG1^+^ gate, the number of tetramer^+^CD8^+^KLRG1^+^ events was also reviewed, and all included samples contained ≥50 events in this downstream gate.

Intracellular IFN-γ and TNF-α were analyzed within the indicated Ag85A_48_-_56_ tetramer^+^CD8^+^ T cell population. Details of the revised cytokine-stimulation and protein-transport-inhibition protocol are provided in Section 2.7.

### 2.7 *In vitro* PBMC stimulation assay

Freshly isolated PBMCs were resuspended in RPMI 1640 medium supplemented with 10% fetal bovine serum and 1% penicillin-streptomycin and seeded in 24-well plates at a density of 1 × 10^6^ cells/mL. Cells were cultured for 48 h under the indicated experimental conditions.

For the β-galactosidase/Ki-67 experiments, PBMCs from three independent patients with PTB were evaluated under eight culture conditions: medium alone (unstimulated control), Ag85A at 5μg/mL, Ag85A at 10μg/mL, Ag85A at 5μg/mL plus polymyxin B (10μg/mL), Ag85A at 10μg/mL plus polymyxin B (10μg/mL), anti-CD3/CD28 stimulation alone, anti-CD3/CD28 plus Ag85A at 5μg/mL, and anti-CD3/CD28 plus Ag85A at 10μg/mL. Polymyxin B (MedChemExpress; Cat. No. HY-149179) was used at a final concentration of 10μg/mL. For anti-CD3/CD28 stimulation, plates were pre-coated with anti-CD3 and anti-CD28 antibodies at 5μg/mL each. After 48 h of culture, senescence-associated β-galactosidase activity and Ki-67 expression were assessed by flow cytometry as described below.

For the revised intracellular cytokine experiments, PBMCs were evaluated under six culture conditions: medium alone (unstimulated control), a PMA/ionomycin positive-control condition, Ag85A at 5μg/mL, Ag85A at 10μg/mL, Ag85A at 5μg/mL plus polymyxin B (10μg/mL), and Ag85A at 10μg/mL plus polymyxin B (10μg/mL). During the final 4 h of the 48-h culture, eBioscience™ Protein Transport Inhibitor Cocktail (500X; Thermo Fisher Scientific; Cat. No. 00-4980-93), containing brefeldin A and monensin, was added to all experimental conditions to inhibit cytokine secretion and permit intracellular accumulation of IFN-γ and TNF-α. PMA and ionomycin were not added to the unstimulated or Ag85A-containing experimental groups. The positive-control condition additionally received eBioscience™ Cell Stimulation Cocktail (500X; Thermo Fisher Scientific; Cat. No. 00-4970-93), containing PMA and ionomycin. Thus, brefeldin A and monensin were applied uniformly across all groups, whereas PMA/ionomycin stimulation was restricted to the separate positive-control condition.

Following culture, cells were harvested and subjected to Ag85A_48_-_56_ tetramer staining, surface-marker staining, fixation/permeabilization, and intracellular IFN-γ and TNF-α staining as described in Section 2.6. Intracellular cytokine signals detected in the Ag85A-containing groups were therefore measured without an additional PMA/ionomycin-mediated polyclonal restimulation step and were interpreted as intracellular cytokine responses under the indicated Ag85A culture conditions.

### 2.8 β-galactosidase activity and Ki-67 analysis

Following 48 h of *in vitro* stimulation, PBMCs were harvested and stained with the HLA-A*02:01-restricted Ag85A_48_-_56_ tetramer and the indicated surface antibodies as described above. Senescence-associated β-galactosidase activity was measured using the Cell Meter™ Cellular Senescence Activity Assay Kit with red fluorescence (AAT Bioquest; Cat. No. 23007) according to the manufacturer’s instructions.

For Ki-67 analysis, cells were fixed and permeabilized using the BD Cytofix/Cytoperm™ Fixation/Permeabilization Kit (BD Biosciences; Cat. No. 554714), followed by intracellular staining with an anti-human Ki-67 antibody (BioLegend; Cat. No. 350525). β-Galactosidase activity and Ki-67 expression were analyzed within Ag85A_48_-_56_ tetramer^+^CD8^+^ T cells. Ki-67 was interpreted as a proliferation-associated readout rather than as a measure of cell viability. Samples were acquired on a BD FACSAria™ III Cell Sorter and analyzed using FlowJo software version 10.8. As an additional quality-control assessment, cell viability after the 48-h culture period was evaluated using BD Horizon™ Fixable Viability Stain 450 (FVS450; BD Biosciences; Cat. No. 562247) under the eight stimulation conditions described above. Three technical replicate measurements were obtained for each condition. These measurements were used descriptively to provide context for interpretation of the short-term stimulation experiments and were not treated as independent biological replicates or subjected to inferential statistical testing.

### 2.9 Statistical analysis

Statistical analyses were performed using SPSS version 25.0 and GraphPad Prism version 10.1.2. The normality of continuous variables was assessed using the Shapiro–Wilk test. Normally distributed continuous variables are presented as the mean ± standard deviation (SD), whereas non-normally distributed variables are presented as the median and interquartile range (IQR). Categorical variables are presented as numbers and percentages.

For comparisons between two independent groups, the unpaired Student’s t-test was used for normally distributed variables, and the Mann–Whitney U test was used for non-normally distributed variables. For comparisons among three or more independent groups, one-way analysis of variance (ANOVA) followed by Tukey’s multiple-comparison test was used for normally distributed data, whereas the Kruskal–Wallis test followed by Dunn’s multiple-comparison test was used for non-normally distributed data. Reported *post hoc* P values were multiplicity-adjusted P values. When the homogeneity-of-variance assumption was violated, Welch’s ANOVA was used, followed by Dunnett’s T3 multiple-comparison test for pairwise comparisons. Reported pairwise P values were multiplicity-adjusted P values.

For age-stratified analyses involving age category and a categorical clinical factor, including clinical group or sputum smear status, two-way ANOVA was used to evaluate the corresponding main effects and interaction. When simple-effects analyses were performed, comparisons among clinical groups within each age stratum and among age strata within each clinical group were conducted using Tukey’s multiple-comparison test. All reported pairwise P values from these analyses were multiplicity-adjusted P values.

For **Figure 2B**, because CD3^+^CD8^+^ and CD3^+^CD8^⁻^ T cell measurements were obtained from the same participants, a two-way repeated-measures ANOVA was performed, with T cell subset treated as the within-subject factor and clinical group as the between-subject factor. Simple-effects comparisons between CD3^+^CD8^+^ and CD3^+^CD8^⁻^ T cell subsets within each clinical group were performed using Šídák’s multiple-comparison adjustment, and multiplicity-adjusted P values were reported.

**Figure 2:**
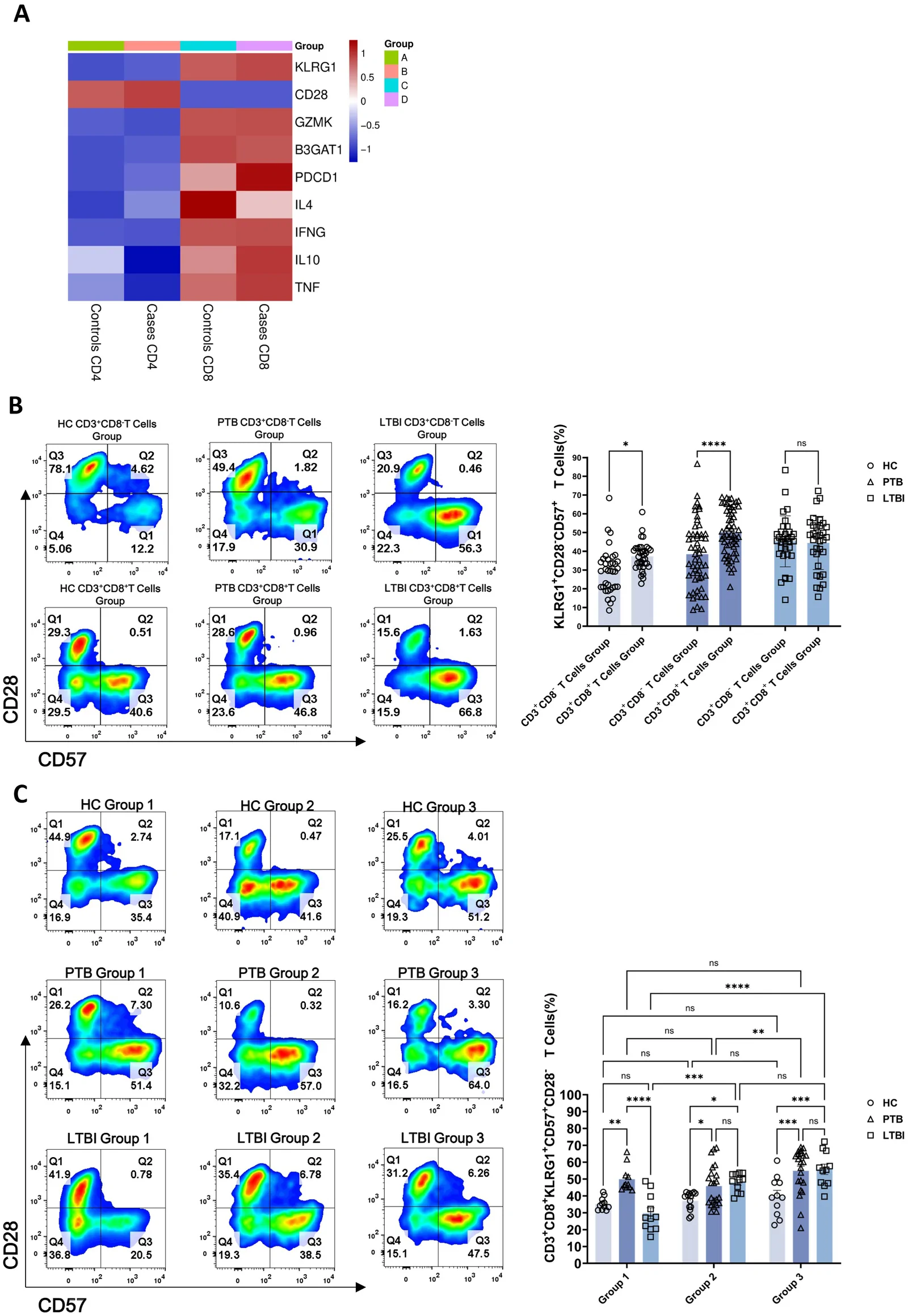
KLRG1^+^CD28^⁻^CD57^+^ cells are enriched within the CD8^+^ T cell compartment in PTB. **(A)** Heatmap showing the relative expression of selected T cell differentiation-, activation-, exhaustion-, and cytokine-related genes in CD4^+^ and CD8^+^ T cell populations from control and tuberculosis case samples in the GSE158769 dataset. The columns labeled “Controls CD4,” “Cases CD4,” “Controls CD8,” and “Cases CD8” represent control CD4^+^ T cells, tuberculosis case CD4^+^ T cells, control CD8^+^ T cells, and tuberculosis case CD8^+^ T cells, respectively. The color scale indicates relative normalized expression. B3GAT1 was included as a transcript-level marker associated with the CD57 epitope. **(B)** Comparison of KLRG1^+^CD28^⁻^CD57^+^ cells between CD3^+^CD8^+^ and CD3^+^CD8^⁻^ T cell subsets. Two-way repeated-measures ANOVA showed significant effects of T cell subset (F(1,121) = 13.09, P = 0.0004) and clinical group (F(2,121) = 16.68, P < 0.0001), together with a significant interaction (F(2,121) = 4.726, P = 0.0106). Šídák-adjusted P values for CD8^+^ versus CD8^⁻^ comparisons were 0.0422 in HCs, <0.0001 in PTB, and 0.9913 in LTBI. ns, not significant; *adjusted P < 0.05; ****adjusted P < 0.0001. **(C)** Age-stratified comparison of KLRG1^+^CD28^⁻^CD57^+^ cells among total CD8^+^ T cells. Two-way ANOVA showed significant effects of age category (F(2,115) = 12.58, P < 0.0001) and clinical group (F(2,115) = 16.67, P < 0.0001), together with a significant interaction (F(4,115) = 5.678, P = 0.0003). Tukey-adjusted P values for PTB versus HC, PTB versus LTBI, and HC versus LTBI were 0.0026, <0.0001, and 0.3947 in Group 1; 0.0346, 0.8313, and 0.0234 in Group 2; and 0.0003, 0.9671, and 0.0009 in Group 3, respectively. Within the PTB group, Group 2 versus Group 3 was significant (P = 0.0100), whereas the other age comparisons were not significant. Within the LTBI group, Group 1 versus Group 2 (P = 0.0001) and Group 1 versus Group 3 (P < 0.0001) were significant. No age-related comparisons were significant within HCs. All pairwise P values are multiplicity-adjusted. ns, not significant; *adjusted P < 0.05; **adjusted P < 0.01; ***adjusted P < 0.001; ****adjusted P < 0.0001.

Categorical variables were compared using the chi-square test or Fisher’s exact test, as appropriate. For *in vitro* experiments in which PBMCs from the same donor were evaluated under multiple stimulation conditions, repeated-measures one-way ANOVA followed by an appropriate multiple-comparison test was used for normally distributed data, whereas the Friedman test followed by Dunn’s multiple-comparison test was used for non-normally distributed data. For the β-galactosidase/Ki-67 experiments, PBMCs from three independent PTB donors were evaluated under all stimulation conditions, with measurements across conditions obtained from the same donors. These data were therefore treated as matched repeated measures. Given the small number of biological replicates, differences across stimulation conditions were evaluated using the Friedman test, followed by Dunn’s multiple-comparison test for prespecified pairwise contrasts. Reported pairwise P values were multiplicity-adjusted P values.

For the intracellular cytokine experiments presented in **Figures 3** and **4**, PBMCs from three independent PTB donors were evaluated under all six stimulation conditions, with measurements across conditions obtained from the same donors. These data were therefore treated as matched repeated measures. Given the small number of biological replicates, overall differences across stimulation conditions were evaluated using the Friedman test, with exact P values as reported by GraphPad Prism, followed by Dunn’s multiple-comparison test for prespecified pairwise contrasts. Reported pairwise P values were multiplicity-adjusted P values.

**Figure 3:**
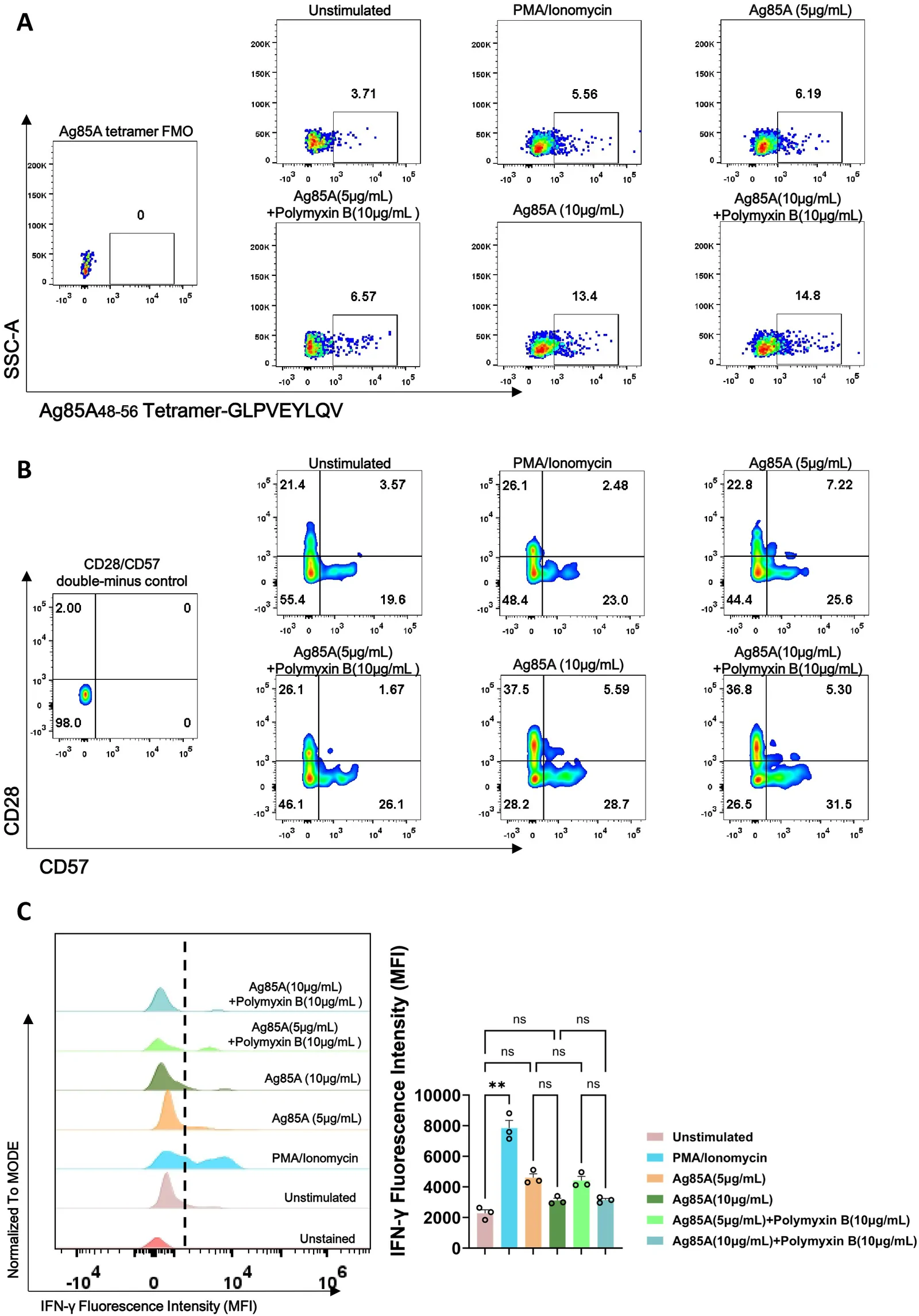
Intracellular IFN-γ responses in Ag85A-specific CD8^+^ T cells under defined *in vitro* stimulation conditions. **(A)** Representative flow-cytometric plots showing HLA-A*02:01-restricted Ag85A_48_-_56_ tetramer staining within CD3^+^CD8^+^ T cells under the indicated culture conditions: unstimulated control, PMA/ionomycin positive control, Ag85A at 5μg/mL, Ag85A at 10μg/mL, Ag85A at 5μg/mL plus polymyxin B (10μg/mL), and Ag85A at 10μg/mL plus polymyxin B (10μg/mL). An Ag85A tetramer fluorescence-minus-one (FMO) control is shown for gate definition. Numbers indicate the percentage of Ag85A_48_-_56_ tetramer^+^ cells within the corresponding CD3^+^CD8^+^ T cell population. **(B)** Representative CD28/CD57 quadrant plots showing CD28 and CD57 expression within KLRG1^+^ Ag85A_48_-_56_ tetramer^+^CD8^+^ T cells under the same culture conditions. A CD28/CD57 double-minus control is shown as a background reference for quadrant placement. Numbers in each quadrant indicate the percentage of cells within the corresponding parent population. **(C)** Representative histogram overlays and quantitative comparison of intracellular IFN-γ mean fluorescence intensity (MFI) in KLRG1^+^CD28^⁻^CD57^+^ Ag85A_48_-_56_ tetramer^+^CD8^+^ T cells under the indicated culture conditions. PBMCs from three independent patients with PTB were analyzed (biological n = 3). During the final 4 h of culture, eBioscience™ Protein Transport Inhibitor Cocktail containing brefeldin A and monensin was added to all experimental conditions, whereas PMA/ionomycin was restricted to the separate positive-control condition. Polymyxin B was used at a final concentration of 10μg/mL in the indicated Ag85A-containing groups. Each data point represents one independent PTB donor. Differences across the six matched stimulation conditions were evaluated using the Friedman test with an exact P value, followed by Dunn’s multiple-comparison test for seven prespecified pairwise contrasts. The overall Friedman test was significant (Friedman statistic = 14.62, exact P < 0.0001). The Dunn-adjusted P value was 0.0074 for the unstimulated versus PMA/ionomycin positive-control comparison, 0.0618 for unstimulated versus Ag85A 5μg/mL, and >0.9999 for unstimulated versus Ag85A 10μg/mL. The remaining prespecified comparisons, including the corresponding Ag85A-only versus polymyxin B-containing conditions and the Ag85A concentration comparison, were not statistically significant after Dunn’s adjustment (all adjusted P > 0.05). All reported pairwise P values are Dunn-adjusted P values. ns, not significant; **adjusted P < 0.01.

**Figure 4:**
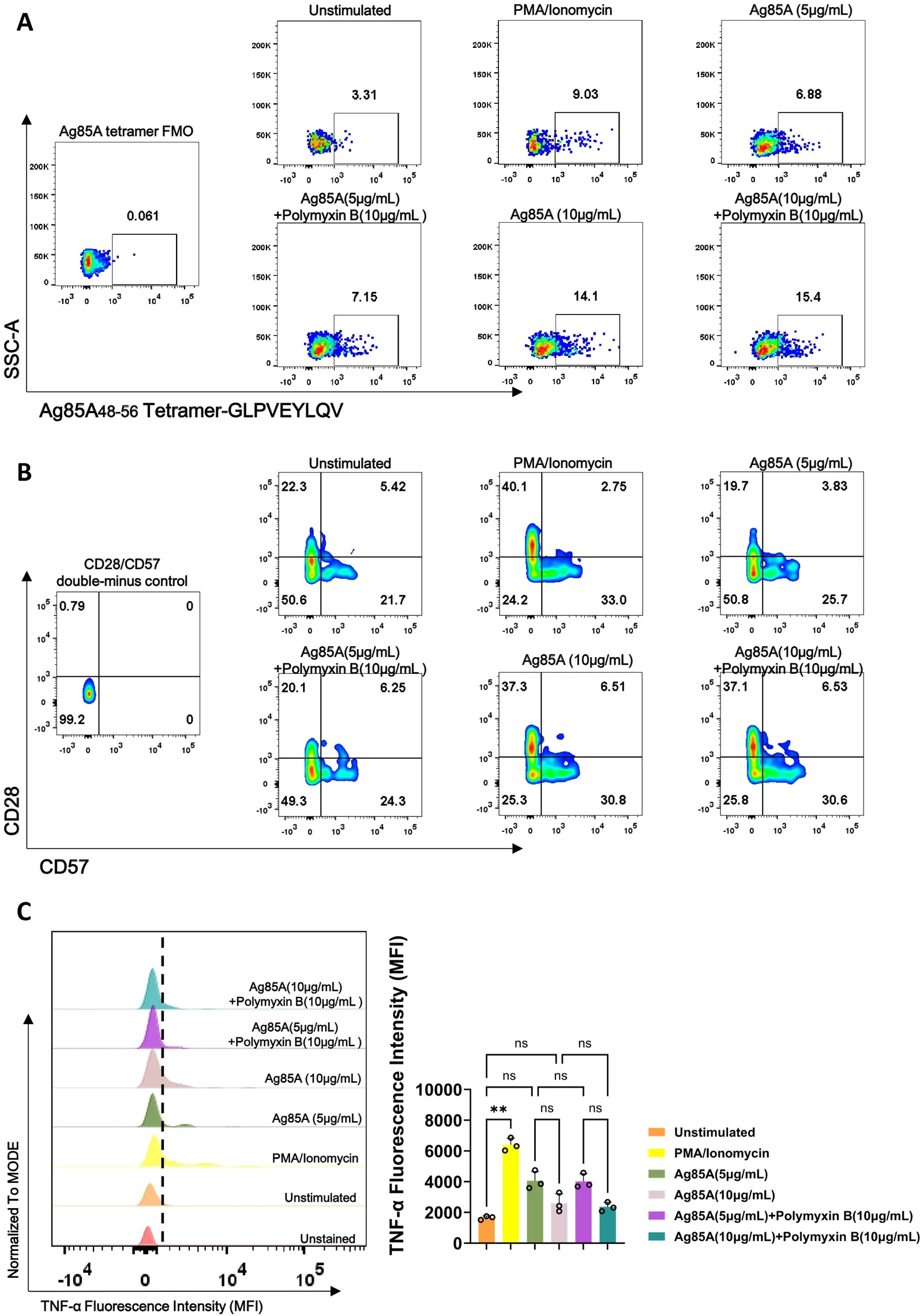
Intracellular TNF-α responses in Ag85A-specific CD8^+^ T cells under revised *in vitro* stimulation conditions. **(A)** Representative flow-cytometric plots showing HLA-A*02:01-restricted Ag85A_48_-_56_ tetramer staining within CD3^+^CD8^+^ T cells under the indicated culture conditions: unstimulated control, PMA/ionomycin positive control, Ag85A at 5μg/mL, Ag85A at 10μg/mL, Ag85A at 5μg/mL plus polymyxin B (10μg/mL), and Ag85A at 10μg/mL plus polymyxin B (10μg/mL). An Ag85A tetramer fluorescence-minus-one (FMO) control is shown for gate definition. Numbers indicate the percentage of Ag85A_48_-_56_ tetramer^+^ cells within the corresponding CD3^+^CD8^+^ T cell population. **(B)** Representative CD28/CD57 quadrant plots showing CD28 and CD57 expression within KLRG1^+^ Ag85A_48_-_56_ tetramer^+^CD8^+^ T cells under the same culture conditions. A CD28/CD57 double-minus control is shown as a background reference for quadrant placement. Numbers in each quadrant indicate the percentage of cells within the corresponding parent population. **(C)** Representative histogram overlays and quantitative comparison of intracellular TNF-α mean fluorescence intensity (MFI) in KLRG1^+^CD28^⁻^CD57^+^ Ag85A_48_-_56_ tetramer^+^CD8^+^ T cells under the indicated culture conditions. PBMCs from three independent patients with PTB were analyzed (biological n = 3). During the final 4 h of culture, eBioscience™ Protein Transport Inhibitor Cocktail containing brefeldin A and monensin was added to all experimental conditions, whereas PMA/ionomycin was restricted to the separate positive-control condition. Polymyxin B was used at a final concentration of 10μg/mL in the indicated Ag85A-containing groups. Each data point represents one independent PTB donor. Differences across the six matched stimulation conditions were evaluated using the Friedman test with an exact P value, followed by Dunn’s multiple-comparison test for seven prespecified pairwise contrasts. The overall Friedman test was significant (Friedman statistic = 14.62, exact P < 0.0001). The Dunn-adjusted P value was 0.0074 for the unstimulated versus PMA/ionomycin positive-control comparison and 0.1146 for unstimulated versus Ag85A 5μg/mL. The unstimulated versus Ag85A 10μg/mL comparison, the corresponding Ag85A-only versus polymyxin B-containing comparisons, and the Ag85A concentration comparison were not statistically significant after Dunn’s adjustment (all adjusted P > 0.05). All reported pairwise P values are Dunn-adjusted P values. ns, not significant; **adjusted P < 0.01.

Associations between the proportion of KLRG1^+^CD28^⁻^CD57^+^ cells among Ag85A_48_-_56_ tetramer^+^CD8^+^ T cells and baseline clinical or laboratory parameters were evaluated using correlation analysis as specified for the corresponding dataset. For the 12 correlation analyses presented in **Figure 5**, the distribution of each clinical or laboratory variable was assessed using the Shapiro–Wilk test. Pearson correlation coefficients (r) were used for approximately normally distributed variables, including neutrophil percentage, lymphocyte percentage, and albumin, whereas Spearman rank correlation coefficients (ρ) were used for skewed or heavy-tailed variables, including monocyte percentage, C-reactive protein (CRP), and the plasma cytokine measurements. The correlation method used for each variable is indicated in the corresponding **Figure 5** panel. Because these 12 tests addressed a common exploratory question, the resulting nominal P values were adjusted as a single testing family using the Benjamini–Hochberg false discovery rate (FDR) procedure. Both nominal P values and FDR-adjusted P values were reported, and an FDR-adjusted P < 0.05 was considered statistically significant after correction for multiple testing.

**Figure 5:**
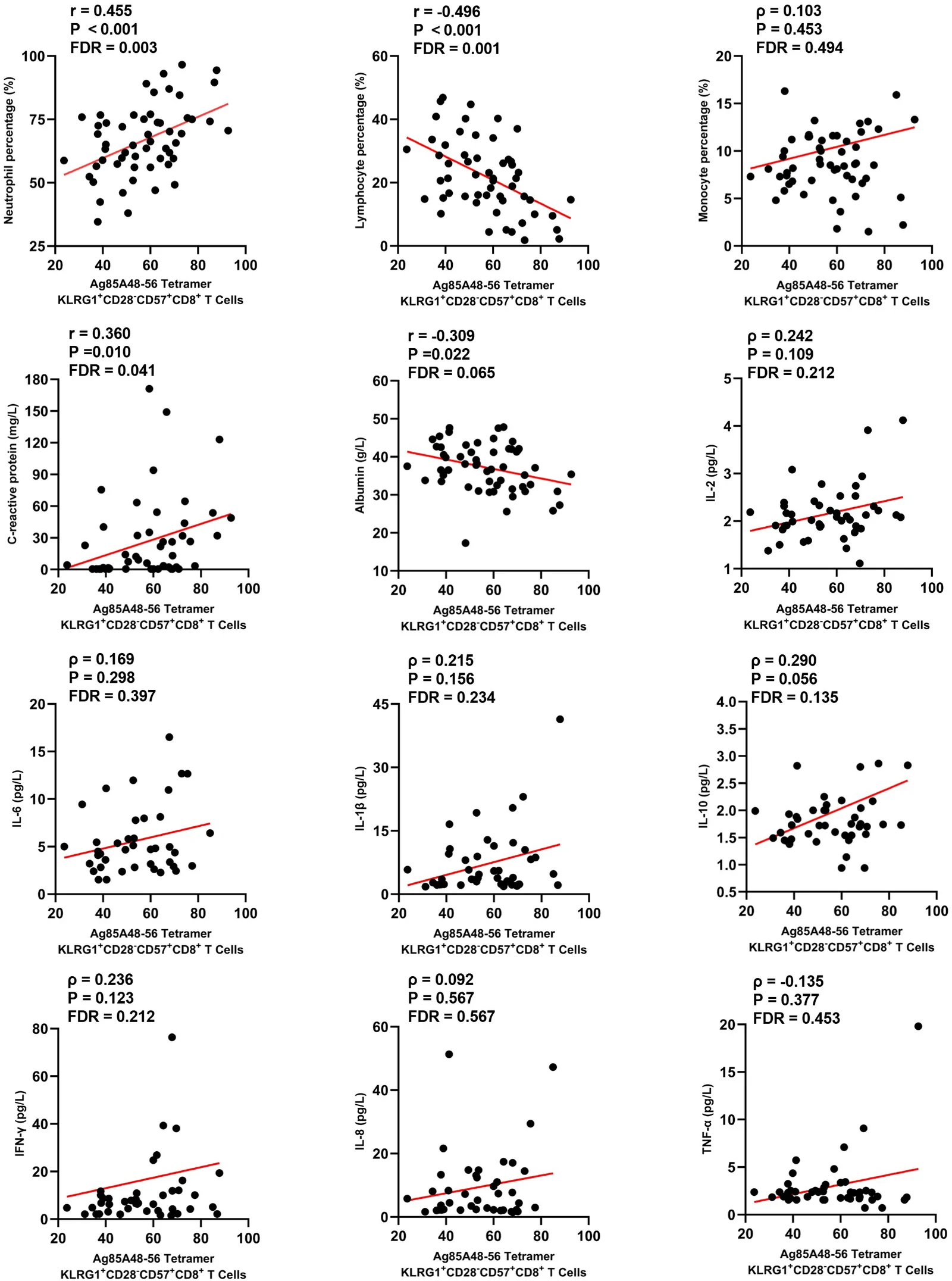
Associations between KLRG1^+^CD28^⁻^CD57^+^ Ag85A-specific CD8^+^ T cells and baseline clinical laboratory parameters in PTB. Correlation analyses were performed between the proportion of KLRG1^+^CD28^⁻^CD57^+^ cells among Ag85A_48_-_56_ tetramer^+^CD8^+^ T cells and 12 selected baseline clinical and laboratory parameters. Each dot represents one patient with PTB. Pearson correlation coefficients (r) were used for approximately normally distributed variables, whereas Spearman rank correlation coefficients (ρ) were used for skewed or heavy-tailed variables. The correlation method, nominal P value, and Benjamini–Hochberg FDR-adjusted P value (P FDR) are indicated in each panel. Significant correlations were observed for neutrophil percentage (r = 0.455, P < 0.001; P FDR = 0.003), lymphocyte percentage (r = −0.496, P < 0.001; P FDR = 0.001), and CRP (ρ = 0.360, P = 0.010; P FDR = 0.041). Albumin showed a nominal inverse correlation (r = −0.309, P = 0.022), but this association did not remain significant after FDR correction (P_FDR = 0.065). Monocyte percentage was not significantly associated with the phenotype (ρ = 0.103, P = 0.453; P FDR = 0.494). None of the examined plasma cytokines remained statistically significant after FDR correction.

The between-group comparisons of clinical, radiological, and laboratory variables presented in **Table 1** and **Supplementary Table S2** were intended primarily for descriptive clinical characterization of the severe and non-severe PTB groups. Because these analyses involved multiple heterogeneous exploratory variables, the corresponding P values are reported as nominal, unadjusted P values and should be interpreted as hypothesis-generating rather than confirmatory. Accordingly, borderline nominal P values were not used as independent evidence supporting the primary study conclusions.

**Table 1:** Selected clinical, radiological, laboratory, and immunophenotypic characteristics of patients with non-severe and severe PTB.

Characteristics	Non-severe PTB group (n=30)	Severe PTB group (n=25)	Nominal P value
Immunophenotypic variable			
Ag85A48–56 Tetramer KLRG1^+^CD28^-^CD57^+^CD8^+^ T Cells (%)	48.91 ± 13.39	66.93 ± 12.23	< 0.001
Clinical Symptoms and Radiological Findings, n (%)			
Cough	16 (53.3%)	22 (88.0%)	0.008
Dyspnea	0 (0.0%)	6 (24.0%)	0.004
Fatigue	1 (3.3%)	9 (36.0%)	< 0.001
Number of involved lung fields	2.00 (1.00, 2.00)	4.00 (4.00, 4.00)	< 0.001
Cavitation	0 (0.0%)	9 (36.0%)	< 0.001
Comorbidities, n (%)			
Diabetes mellitus	2 (6.7%)	6 (24.0%)	0.058
Respiratory failure	0 (0.0%)	6 (24.0%)	0.005
Laboratory Parameters			
Albumin (g/L)	39.30 ± 4.35	34.61 ± 7.38	0.005
C-reactive protein (mg/L)	1.60 (0.50, 11.45)	14.30 (2.30, 31.92)	0.044
Lymphocyte percentage (%)	26.50 ± 10.80	17.18 ± 10.23	0.002
Hemoglobin (g/L)	124.10 ± 14.19	109.96 ± 25.49	0.012
IFN-α (pg/L)	1.86 (1.56, 2.02)	1.54 (1.32, 1.85)	0.11
IL-6 (pg/L)	4.71 (3.09, 6.82)	5.70 (3.23, 11.71)	0.191
IFN-γ (pg/L)	6.57 (3.90, 8.65)	12.00 (4.24, 26.36)	0.102

Clinical characteristics, laboratory findings, chest CT features, and the proportion of KLRG1^+^CD28^⁻^CD57^+^ Ag85A-specific CD8^+^ T cells in patients with severe versus non-severe pulmonary tuberculosis. Data normality was assessed with the Shapiro–Wilk test. Normally distributed continuous variables are presented as mean ± standard deviation (SD), whereas non-normally distributed data are summarized as median (interquartile range, IQR). Categorical variables are reported as number (percentage), n (%). Comparisons of clinical, radiological, and laboratory variables in this table were exploratory and are provided primarily for descriptive characterization of the severe and non-severe PTB groups. Reported P values are nominal and were not adjusted for multiple comparisons. Therefore, these P values should be interpreted as hypothesis-generating rather than confirmatory, particularly for values close to the conventional P = 0.05 threshold.

Univariable logistic regression analysis was performed to identify variables associated with severe PTB. Given the limited size of the PTB cohort (n = 55) and the number of severe PTB events (n = 25), a parsimonious modeling strategy was adopted to reduce the risk of overfitting. Variables associated with severe PTB at P < 0.05 in univariable analysis were entered into the primary multivariable logistic regression model. The number of covariates was therefore deliberately restricted rather than simultaneously including all potentially relevant clinical variables. Results are reported as odds ratios (ORs) with 95% confidence intervals (CIs). These regression analyses were considered exploratory.

Receiver operating characteristic (ROC) curve analysis was performed to assess the exploratory in-cohort discriminative potential of the proportion of KLRG1^+^CD28^⁻^CD57^+^ cells among Ag85A_48_-_56_ tetramer^+^CD8^+^ T cells for distinguishing severe from non-severe PTB. The apparent area under the ROC curve (AUC), 95% CI, sensitivity, specificity, and exploratory cutoff value were calculated. Internal stability of the AUC estimate was assessed using patient-level bootstrap resampling with 10,000 iterations. Bootstrap mean and median AUCs, bootstrap 95% CIs, and bootstrap bias-corrected AUCs were calculated to assess the internal stability and bootstrap-estimated bias of the apparent AUC estimate.

To assess whether the severity-associated findings were influenced by low antigen-specific event counts, an additional sensitivity analysis was performed using a conservative minimum of ≥100 Ag85A_48_-_56_ tetramer^+^CD8^+^ T cell events. The severe-versus-non-severe PTB comparison and the corresponding ROC analysis were reassessed after application of this criterion. Because all 55 PTB samples in the final analytic cohort contained ≥100 tetramer^+^CD8^+^ events, no PTB samples were excluded and the event-count-restricted analyses were identical to the primary analyses.

As complementary sensitivity analyses, the association between the KLRG1^+^CD28^⁻^CD57^+^ Ag85A-specific CD8^+^ T cell proportion and disease severity was evaluated using TB score and CT score. Both scores were analyzed as continuous variables using Spearman rank correlation. In addition, the exploratory ROC analysis was repeated using the established TB score-based clinical severity definition (TB score ≥8 vs <8). Because these analyses were performed within the same cohort used for marker evaluation, they were considered sensitivity analyses and hypothesis-generating rather than evidence of externally validated diagnostic or prognostic performance.

All statistical tests were two-sided. For *post hoc* pairwise comparisons following ANOVA or nonparametric omnibus tests, multiplicity-adjusted P values were reported using the corresponding multiple-comparison procedures.

## 3 Results

### 3.1 Clinical characteristics of the study participants and age-stratified frequencies of Ag85A-specific CD8^+^ T cells

A total of 192 candidates were initially screened, including 92 PTB candidates, 45 LTBI candidates, and 55 HC candidates. During participant selection, 52 candidates were excluded because of absent or insufficient detectable Ag85A_48_-_56_ tetramer^+^CD8^+^ T cell events for reliable downstream phenotypic analysis, including 21 PTB, 12 LTBI, and 19 HC candidates. An additional 16 PTB candidates were excluded because of incomplete clinical data. After these exclusions, 124 participants were included in the final analytic cohort, comprising 55 patients with PTB, 33 individuals with LTBI, and 36 HCs (**Figure 1A**).

The PTB cohort included 30 males and 25 females, with a median age of 34 years (IQR, 24–52 years). The LTBI cohort included 16 males and 17 females, with a median age of 32.5 years (IQR, 14–47.5 years). The HC group included 18 males and 18 females, with a median age of 31.5 years (IQR, 14–55 years). No significant difference in age was observed among the three groups (P = 0.408) (**Supplementary Table S1**; **Figure 1A**).

According to the predefined clinical and radiological criteria described in the Methods, the PTB cohort included 30 patients with non-severe disease (54.5%) and 25 patients with severe disease (45.5%) (Yu et al., 2022; Yu et al., 2024) (**Table 1**).

Based on previously reported age-related changes in thymic function, participants in the HC, LTBI, and PTB groups were stratified into three predefined age categories: Group 1, ≤12 years; Group 2, 13–39 years; and Group 3, ≥40 years (Deng et al., 2025). Flow-cytometric analyses were performed according to the gating strategy shown in **Figure 1B**, and representative plots of tetramer-defined Ag85A-specific CD8^+^ T cells are shown in **Figure 6A**.

**Figure 6:**
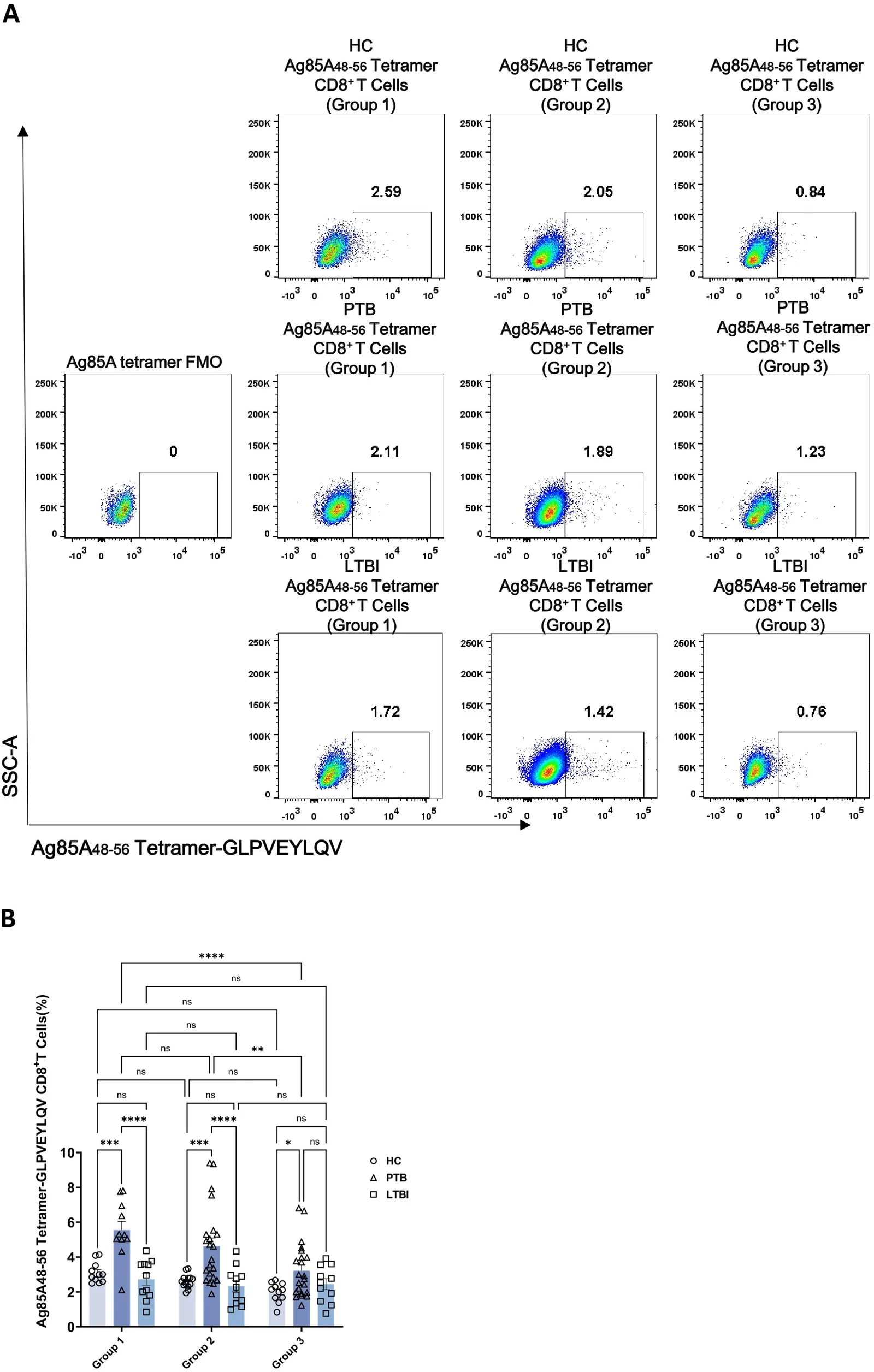
Age-stratified frequencies of Ag85A_48_-_56_ tetramer^+^CD8^+^ T cells in healthy controls, patients with active pulmonary tuberculosis, and individuals with latent tuberculosis infection. **(A)** Representative flow-cytometric plots showing Ag85A_48_-_56_ tetramer staining within the CD8^+^ T-cell population from healthy controls (HCs), patients with active pulmonary tuberculosis (PTB), and individuals with latent tuberculosis infection (LTBI) across three predefined age groups. An Ag85A_48_-_56_ tetramer fluorescence-minus-one (FMO) control is shown in the left column as a reference for definition of the tetramer-positive gate. Ag85A-specific CD8^+^ T cells were identified using an HLA-A*02:01-restricted Ag85A_48_-_56_ tetramer containing the GLPVEYLQV peptide. Numbers in the plots indicate the percentage of Ag85A_48_-_56_ tetramer^+^ cells within the corresponding CD8^+^ T cell gate. **(B)** Age-stratified comparison of Ag85A_48_-_56_ tetramer^+^CD8^+^ T cell frequencies among HCs, patients with PTB, and individuals with LTBI. Two-way ANOVA showed significant main effects of clinical group (F(2,115) = 27.80, P < 0.0001) and age category (F(2,115) = 7.268, P = 0.0011), whereas the clinical-group-by-age interaction was not significant (F(4,115) = 2.030, P = 0.0948). Tukey-adjusted P values for HC versus PTB, HC versus LTBI, and PTB versus LTBI were 0.0002, 0.8069, and <0.0001 in Group 1; 0.0001, 0.8695, and <0.0001 in Group 2; and 0.0462, 0.7209, and 0.2875 in Group 3, respectively. Within the PTB group, adjusted P values were 0.1739 for Group 1 versus Group 2, <0.0001 for Group 1 versus Group 3, and 0.0030 for Group 2 versus Group 3. No age-related comparisons were significant within the HC or LTBI groups. All pairwise P values are Tukey-adjusted. ns, not significant; *adjusted P < 0.05; **adjusted P < 0.01; ***adjusted P < 0.001; ****adjusted P < 0.0001.

Two-way ANOVA showed significant main effects of clinical group and age category, whereas the clinical-group-by-age interaction was not statistically significant. Across age strata, patients with PTB generally exhibited higher frequencies of Ag85A_48_-_56_ tetramer^+^CD8^+^ T cells than HCs. PTB patients also showed higher frequencies than individuals with LTBI in Groups 1 and 2, whereas no significant PTB-versus-LTBI difference was observed in Group 3. No significant differences between HCs and individuals with LTBI were detected within any age stratum. Within the PTB group, Ag85A_48_-_56_ tetramer^+^CD8^+^ T cell frequencies were lower in Group 3 than in Groups 1 and 2 (**Figure 6B**).

### 3.2 A terminal differentiation-associated phenotype is enriched in the CD8^+^ T cell compartment in PTB

Previous studies have used KLRG1 and CD57 as markers associated with T-cell terminal differentiation and immunosenescence (Simpson et al., 2010; Orillard et al., 2023). Analysis of the GSE158769 dataset showed that transcripts associated with KLRG1 and the CD57-related marker B3GAT1 were enriched in the CD8^+^ T cell compartment (**Figure 2A**). Based on this observation, we further examined terminal differentiation-associated T cell phenotypes in clinical samples using flow cytometry.

Participants in the HC, LTBI, and PTB groups were analyzed according to the predefined age categories of ≤12 years, 13–39 years, and ≥40 years (Deng et al., 2025). Flow-cytometric analyses were performed according to the gating strategy shown in **Figure 1B**. For the purposes of this study, the KLRG1^+^CD28^⁻^CD57^+^ phenotype was used operationally to identify a highly differentiated, terminal differentiation-associated T cell population (Zhao et al., 2020; Orillard et al., 2023; Zhang et al., 2025).

A two-way repeated-measures ANOVA showed significant effects of T cell subset and clinical group, together with a significant interaction. The proportion of KLRG1^+^CD28^⁻^CD57^+^ cells was higher in CD3^+^CD8^+^ than in CD3^+^CD8^⁻^ T cells in HCs and patients with PTB, whereas no significant difference was observed in individuals with LTBI (**Figure 2B**). Subsequent analyses therefore focused on the CD8^+^ T cell compartment.

Two-way ANOVA showed significant effects of age category and clinical group, together with a significant age-category-by-clinical-group interaction. Within Group 1, patients with PTB showed higher proportions of KLRG1^+^CD28^⁻^CD57^+^ cells among total CD8^+^ T cells than both HCs and individuals with LTBI. Within Groups 2 and 3, both patients with PTB and individuals with LTBI showed higher proportions than HCs, whereas PTB and LTBI did not differ significantly. Age-related differences were observed within the PTB and LTBI groups, but not among HCs (**Figure 2C**).

### 3.3 The KLRG1^+^CD28^⁻^CD57^+^ phenotype is enriched within Ag85A-specific CD8^+^ T cells in active PTB

The terminal differentiation-associated phenotype of tetramer-defined Ag85A-specific CD8^+^ T cells was evaluated by flow cytometry in patients with PTB, individuals with LTBI, and HCs (**Figure 7A**). In this study, KLRG1^+^CD28^⁻^CD57^+^ Ag85A-specific CD8^+^ T cells were defined as KLRG1^+^CD28^⁻^CD57^+^ cells within the Ag85A_48_-_56_ tetramer^+^CD8^+^ T cell gate.

**Figure 7:**
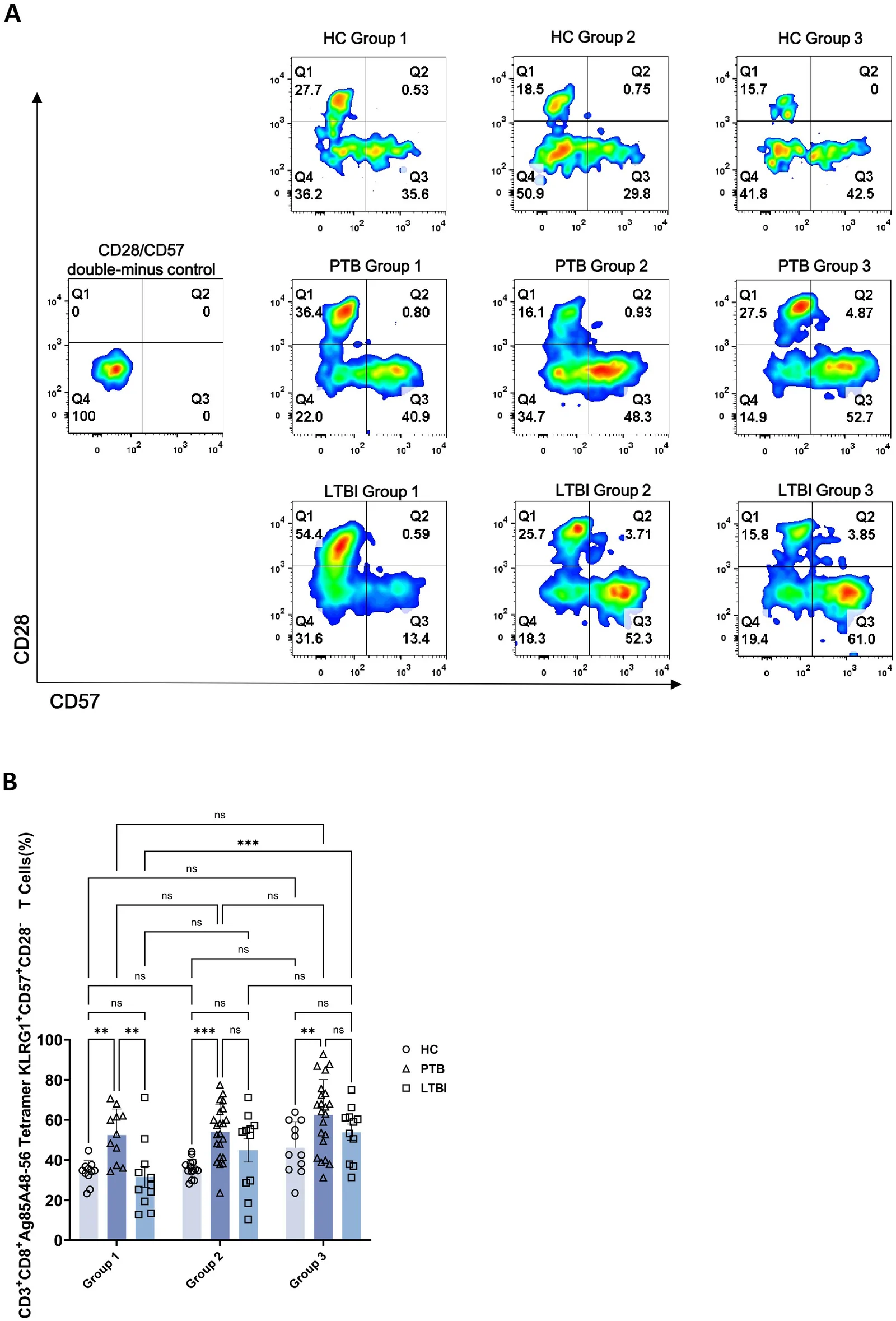
KLRG1^+^CD28^⁻^CD57^+^ cells are enriched within Ag85A-specific CD8^+^ T cells in PTB. **(A)** Representative flow-cytometric CD28/CD57 quadrant plots showing the distribution of CD28 and CD57 expression within KLRG1^+^ Ag85A_48_-_56_ tetramer^+^CD8^+^ T cells from healthy controls (HCs), patients with pulmonary tuberculosis (PTB), and individuals with latent tuberculosis infection (LTBI) across three predefined age groups. A CD28/CD57 double-minus control, rather than separate CD28 and CD57 FMO controls, is shown in the left column as a background reference for quadrant placement. Ag85A-specific CD8^+^ T cells were identified using an HLA-A*02:01-restricted Ag85A_48_-_56_ tetramer, and the KLRG1^+^CD28^⁻^CD57^+^ subset was defined by KLRG1 positivity together with loss of CD28 and expression of CD57 within the tetramer-positive CD8^+^ T cell population. Numbers in the quadrants indicate the percentage of cells within the corresponding parent gate. **(B)** Age-stratified comparison of KLRG1^+^CD28^⁻^CD57^+^ Ag85A-specific CD8^+^ T cells among HCs, patients with PTB, and individuals with LTBI. Two-way ANOVA showed significant main effects of clinical group (F(2,115) = 18.05, P < 0.0001) and age category (F(2,115) = 10.35, P < 0.0001), whereas the clinical-group-by-age interaction was not significant (F(4,115) = 0.8689, P = 0.4850). Tukey-adjusted P values for PTB versus HC, PTB versus LTBI, and HC versus LTBI were 0.0071, 0.0021, and 0.9220 in Group 1; 0.0007, 0.1948, and 0.2432 in Group 2; and 0.0062, 0.2327, and 0.4032 in Group 3, respectively. No significant age-related differences were observed within the HC or PTB groups. Within the LTBI group, Group 3 showed a higher proportion than Group 1 (adjusted P = 0.0010), whereas Group 1 versus Group 2 (adjusted P = 0.0728) and Group 2 versus Group 3 (adjusted P = 0.3011) were not significant. All pairwise P values are Tukey-adjusted. ns, not significant; *adjusted P < 0.05; **adjusted P < 0.01; ***adjusted P < 0.001; ****adjusted P < 0.0001.

Two-way ANOVA showed significant main effects of clinical group and age category, whereas the clinical-group-by-age interaction was not statistically significant. Across all three age strata, patients with PTB showed higher proportions of KLRG1^+^CD28^⁻^CD57^+^ Ag85A-specific CD8^+^ T cells than HCs. PTB patients also showed a higher proportion than individuals with LTBI in Group 1, whereas no significant PTB-versus-LTBI differences were observed in Groups 2 or 3. No significant differences between HCs and individuals with LTBI were observed within any age stratum. Age-related differences were detected within the LTBI group but not within the HC or PTB groups (**Figure 7B**). Together, these findings indicate enrichment of this terminal differentiation-associated phenotype in active PTB across age strata.

### 3.4 KLRG1^+^CD28^⁻^CD57^+^ Ag85A-specific CD8^+^ T cells are associated with smear-defined bacillary burden and selected host-status parameters

Two-way ANOVA showed a significant main effect of sputum smear status, whereas neither age category nor the age-by-smear-status interaction was statistically significant. Smear-positive patients showed higher proportions of KLRG1^+^CD28^⁻^CD57^+^ Ag85A-specific CD8^+^ T cells than smear-negative patients across all three age strata, with no significant age-related differences within either smear-status subgroup (**Figures 8A, B**).

**Figure 8:**
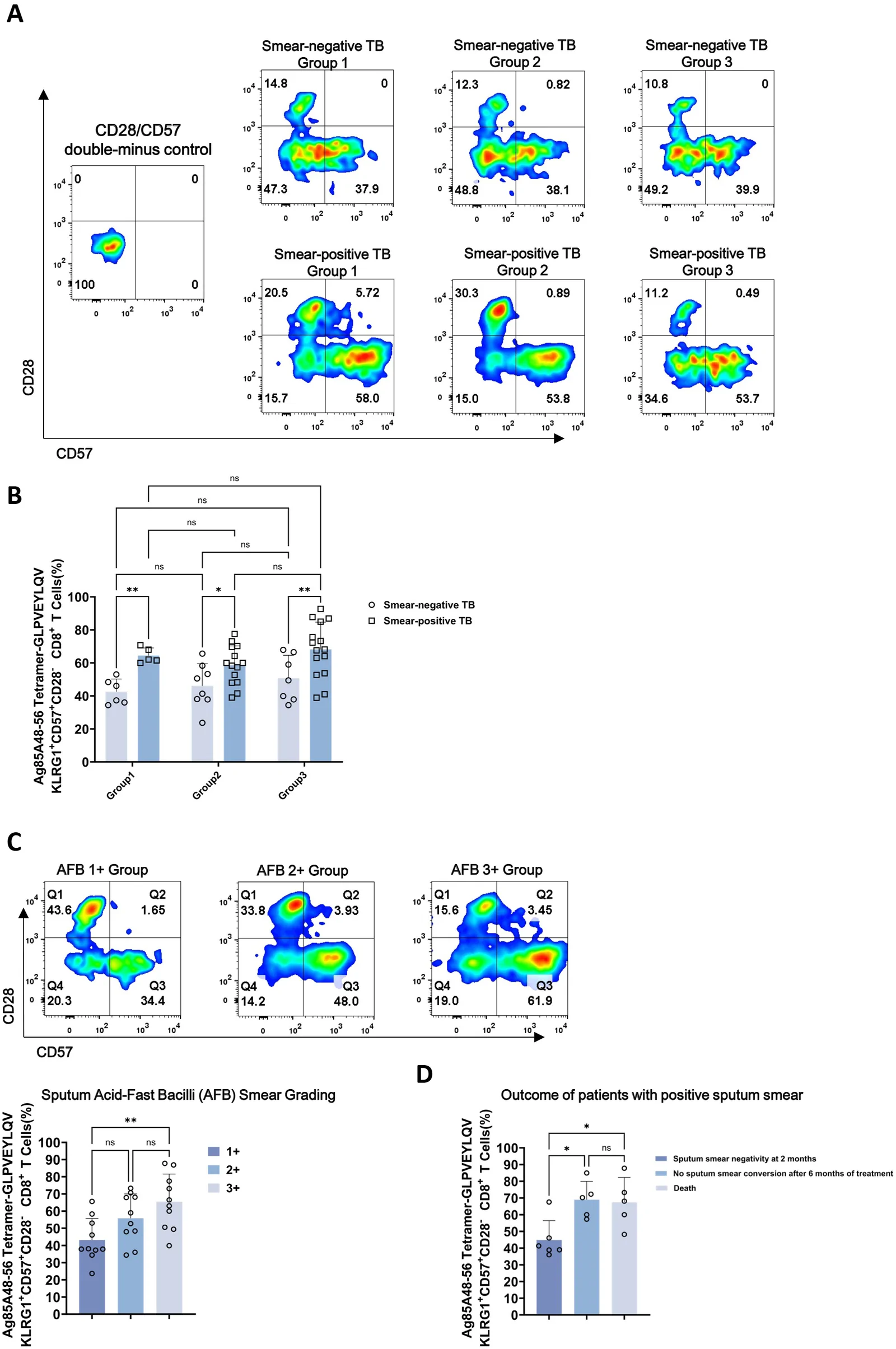
KLRG1^+^CD28^⁻^CD57^+^ Ag85A-specific CD8^+^ T cells in relation to smear-defined bacillary burden and preliminary treatment-related follow-up observations. **(A)** Representative CD28/CD57 quadrant plots showing the distribution of CD28 and CD57 expression within the indicated Ag85A_48_-_56_ tetramer^+^KLRG1^+^CD8^+^ T cell population from sputum smear-negative and sputum smear-positive patients with tuberculosis across three predefined age groups. A CD28/CD57 double-minus control is shown in the left column as a background reference for CD28/CD57 quadrant placement. **(B)** Two-way ANOVA showed a significant main effect of sputum smear status (F(1,49) = 20.28, P < 0.0001), whereas neither age category (F(2,49) = 1.588, P = 0.2147) nor the age-by-smear-status interaction (F(2,49) = 0.4887, P = 0.6164) was significant. Tukey-adjusted P values for smear-positive versus smear-negative comparisons were 0.0080, 0.0369, and 0.0054 in Groups 1, 2, and 3, respectively. **(C)** One-way ANOVA showed a significant overall difference among AFB smear grades (F(2,27) = 6.051, P = 0.0067). Tukey-adjusted P values were 0.1396 for 1+ versus 2+, 0.0049 for 1+ versus 3+, and 0.3067 for 2+ versus 3 +. **(D)** A significant overall difference was observed by the Kruskal–Wallis test (H = 7.965, P = 0.0103). Dunn-adjusted P values were 0.0483 for 2-month smear conversion versus persistent smear positivity, 0.0483 for 2-month smear conversion versus death, and >0.9999 for persistent smear positivity versus death.

When patients were further stratified by sputum AFB smear grade, a significant overall difference was observed among the 1+, 2+, and 3+ groups. The proportion of KLRG1^+^CD28^⁻^CD57^+^ Ag85A-specific CD8^+^ T cells was significantly higher in the AFB 3+ group than in the AFB 1+ group, whereas the 1+ versus 2+ and 2+ versus 3+ comparisons were not statistically significant (**Figure 8C**). These findings support an association between enrichment of this phenotype and higher smear-defined bacillary burden.

To provide preliminary descriptive follow-up context, baseline KLRG1^+^CD28^⁻^CD57^+^ Ag85A-specific CD8^+^ T cell proportions were compared across the small groups of sputum smear-positive patients defined by subsequent follow-up status. A significant overall difference was observed among the three groups. Patients who achieved sputum smear conversion after 2 months had lower baseline proportions than both patients who remained smear-positive after 6 months and patients who died during follow-up, whereas no significant difference was observed between the persistent smear-positive and death groups (**Figure 8D**). Because these follow-up groups were small, these findings are descriptive and should not be interpreted as evidence of prognostic prediction.

Associations between KLRG1^+^CD28^⁻^CD57^+^ Ag85A-specific CD8^+^ T cells and baseline clinical and laboratory indices were examined using Pearson or Spearman correlation, as appropriate. Before multiple-testing correction, significant associations were observed with neutrophil percentage, lymphocyte percentage, CRP, and albumin. After Benjamini–Hochberg FDR correction, the associations with neutrophil percentage, lymphocyte percentage, and CRP remained statistically significant, whereas the association with albumin did not. None of the examined plasma cytokines remained significantly associated with the phenotype after FDR correction (**Figure 5**).

### 3.5 KLRG1^+^CD28^⁻^CD57^+^ Ag85A-specific CD8^+^ T cells mark a severity-associated terminal-differentiation imprint in PTB

According to the predefined clinical and radiological criteria described above, patients with PTB were classified into severe and non-severe groups (**Figure 1A**; **Supplementary Tables S1**, **S2**; **Table 1**).

The clinical, radiological, and laboratory comparisons summarized in **Table 1** and **Supplementary Table S2** were exploratory and descriptive. Because multiple variables were examined, their nominal P values were not interpreted as confirmatory evidence of independent associations with severity; in particular, borderline findings such as the CRP difference (nominal P = 0.044) were not emphasized in the interpretation of the primary results.

The proportion of KLRG1^+^CD28^⁻^CD57^+^ Ag85A-specific CD8^+^ T cells differed significantly among patients with severe PTB, patients with non-severe PTB, individuals with LTBI, and HCs. Patients with severe PTB showed higher proportions than all other groups, whereas patients with non-severe PTB also showed higher proportions than HCs. No significant differences were observed between non-severe PTB and LTBI or between LTBI and HCs (**Figures 9A, B**).

**Figure 9:**
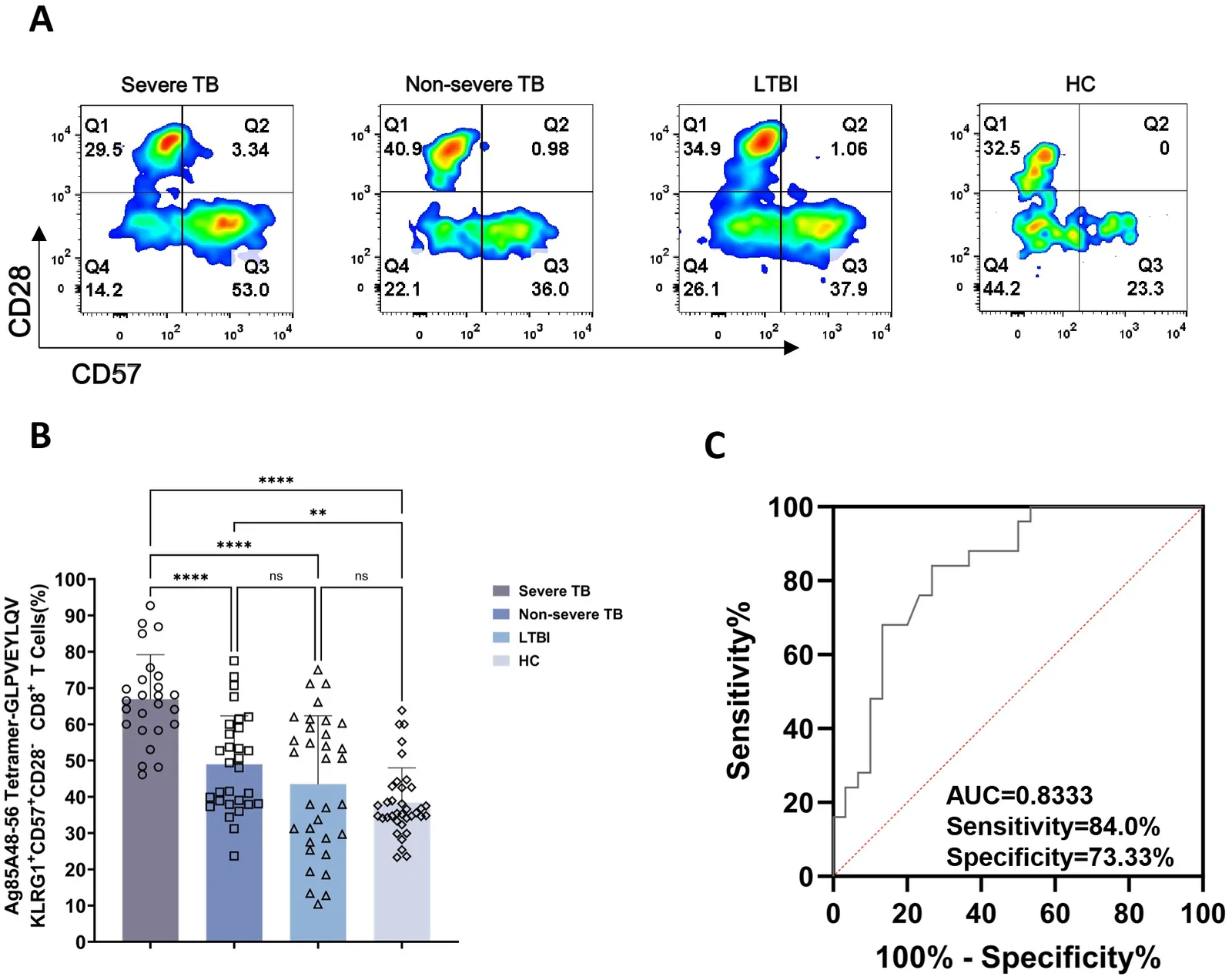
A severity-associated Ag85A-specific CD8^+^ T cell differentiation state in PTB. **(A)** Representative CD28/CD57 quadrant plots within KLRG1^+^ Ag85A_48_-_56_ tetramer^+^CD8^+^ T cells in patients with severe pulmonary tuberculosis (severe PTB), patients with non-severe pulmonary tuberculosis (non-severe PTB), individuals with latent tuberculosis infection (LTBI), and healthy controls (HCs). The analyzed population represents Ag85A_48_-_56_ tetramer^+^CD8^+^ T cells, within which KLRG1 expression and CD28/CD57 co-expression were evaluated. Numbers in the quadrants indicate the percentage of cells within the corresponding parent gate. **(B)** Quantitative comparison of KLRG1^+^CD28^⁻^CD57^+^ Ag85A-specific CD8^+^ T cells among severe PTB, non-severe PTB, LTBI, and HC groups. Because the homogeneity-of-variance assumption was violated, differences among the four groups were analyzed using Welch’s ANOVA, which showed a significant overall difference (W(3, 62.12) = 31.72, P < 0.0001), followed by Dunnett’s T3 multiple-comparison test. The Dunnett’s T3-adjusted P values were <0.0001 for severe PTB versus non-severe PTB, <0.0001 for severe PTB versus LTBI, <0.0001 for severe PTB versus HC, 0.0040 for non-severe PTB versus HC, 0.7043 for non-severe PTB versus LTBI, and 0.6527 for LTBI versus HC. All pairwise P values reported for panel B are Dunnett’s T3-adjusted P values. Data are presented as individual values with summary bars. ns, not significant; **adjusted P < 0.01; ****adjusted P < 0.0001. **(C)** Exploratory receiver operating characteristic (ROC) curve analysis evaluating the in-cohort discriminative potential of KLRG1^+^CD28^⁻^CD57^+^ Ag85A-specific CD8^+^ T cells for distinguishing severe PTB from non-severe PTB. The apparent AUC was 0.8333 (95% CI, 0.7268–0.9398; P < 0.0001). At an exploratory cutoff of 57.80%, sensitivity was 84.0% and specificity was 73.3%. This ROC analysis was exploratory and was not externally validated.

In exploratory logistic regression analyses, the proportion of KLRG1^+^CD28^⁻^CD57^+^ Ag85A-specific CD8^+^ T cells, lymphocyte percentage, and albumin were associated with severe PTB in univariable analysis. In the parsimonious multivariable model, a higher proportion of KLRG1^+^CD28^⁻^CD57^+^ Ag85A-specific CD8^+^ T cells remained associated with severe PTB after adjustment for lymphocyte percentage and albumin (OR = 1.115, 95% CI, 1.038–1.197; P = 0.003) (**Tables 2**, **3**).

**Table 2:** Univariable logistic regression analysis of variables associated with severe PTB.

Variables	β	SE	Wald χ²	P value	OR (95% CI)
Ag85A48–56 Tetramer KLRG1^+^CD28^-^CD57^+^CD8^+^ T Cells (%)	0.108	0.030	13.00	<0.001	1.114 (1.051-1.181)
White blood cell count	0.067	0.075	0.79	0.374	1.069 (0.923-1.238)
Lymphocyte percentage	-0.086	0.031	7.88	0.005	0.918 (0.864-0.974)
Monocyte percentage	0.042	0.052	0.65	0.419	1.043 (0.941-1.156)
Neutrophil percentage	0.034	0.022	2.48	0.115	1.035 (0.992-1.080)
Albumin	-0.138	0.054	6.66	0.010	0.871 (0.784-0.967)
Globulin	0.019	0.037	0.26	0.612	1.019 (0.948-1.095)
Albumin-to-globulin ratio	-0.936	0.744	1.59	0.208	0.392 (0.091-1.684)
C-reactive protein	0.0114	0.0119	0.9253	0.3361	1.012 (0.988-1.035)
IL-10	0.7513	0.6628	1.2847	0.257	2.120 (0.578-7.771)
TNF-α	0.1127	0.1102	1.0467	0.3063	1.119 (0.902-1.389)
IFN-α	-0.4725	0.5168	0.8358	0.3606	0.623 (0.226-1.717)
IL-5	-0.2357	0.3687	0.4087	0.5226	0.790 (0.383-1.627)
IL-6	0.0014	0.0028	0.254	0.6143	1.001 (0.996-1.007)
IFN-γ	-0.0015	0.0035	0.2008	0.6541	0.998 (0.992-1.005)
IL-4	-0.6369	1.7141	0.1381	0.7102	0.529 (0.018-15.222)
IL-2	-0.0401	0.6062	0.0044	0.9473	0.961 (0.293-3.152)
IL-17	0.712	0.4005	3.16	0.0755	2.038 (0.930-4.468)
IL-1β	0.0803	0.0487	2.7244	0.0988	1.084 (0.985-1.192)
IL-12P70	0.8398	0.5747	2.1349	0.144	2.316 (0.751-7.143)
IL-8	0.0091	0.0069	1.7381	0.1874	1.009 (0.996-1.023)

Univariable logistic regression analyses were performed for the following variables: the proportion of KLRG1^+^CD28^⁻^CD57^+^ Ag85A-specific CD8^+^ T cells, lymphocyte percentage, neutrophil percentage, albumin, and other relevant indices.

**Table 3:** Multivariable logistic regression analysis of factors associated with disease severity in PTB patients.

Variables	β	SE	Wald χ²	P value	OR (95% CI)
Ag85A48–56 Tetramer KLRG1^+^CD28^-^CD57^+^CD8^+^ T Cells (%)	0.109	0.036	8.99	0.003	1.115 (1.038 - 1.197)
					
Lymphocyte percentage	0.010	0.043	0.05	0.822	1.010 (0.927 - 1.100)
					
Albumin	-0.111	0.072	2.41	0.120	0.895 (0.777 - 1.030)

Variables with P < 0.05 in the univariable logistic regression analysis—including the proportion of KLRG1^+^CD28^⁻^CD57^+^ Ag85A-specific CD8^+^ T cells, lymphocyte percentage, and albumin—were entered into a multivariable logistic regression model.

ROC analysis showed exploratory in-cohort discrimination between severe and non-severe PTB, with an apparent AUC of 0.8333 (95% CI, 0.7268–0.9398; P < 0.0001). At an exploratory cutoff of 57.80%, sensitivity was 84.0% and specificity was 73.3% (**Figure 9C**). Patient-level bootstrap resampling yielded a bias-corrected AUC of 0.8333, supporting the internal stability of the estimate while not substituting for external validation.

Because all 55 PTB samples contained ≥100 Ag85A_48_-_56_ tetramer^+^CD8^+^ T cell events, application of the conservative ≥100-event sensitivity criterion did not alter the composition of the PTB cohort; consequently, the severe-versus-non-severe comparison and the corresponding ROC analysis were unchanged.

We next examined whether the severity-associated finding was robust when benchmarked against complementary clinical and radiological severity measures. Patients classified as having severe PTB according to the predefined study-specific criteria had a median TB score of 8.0 (IQR, 7.0–9.0; range, 5–12), compared with 2.0 (IQR, 1.0–3.0; range, 0–4) in patients classified as having non-severe PTB. Among the 52 patients with available CT score data, the median CT score was 93.75 (IQR, 75.41–105.00; range, 25–140) in patients classified as having severe PTB (n = 22), compared with 12.5 (IQR, 12.5–28.13; range, 4.17–100) in those classified as having non-severe PTB (n = 30).

The proportion of KLRG1^+^CD28^⁻^CD57^+^ cells among Ag85A_48_-_56_ tetramer^+^CD8^+^ T cells was positively correlated with both TB score (Spearman ρ = 0.5662, P < 0.0001) and CT score (Spearman ρ = 0.5413, P < 0.0001) (**Supplementary Figures S1B, D**).

When clinical severity was alternatively defined using the established TB score threshold of ≥8 versus <8, 19 patients were classified as TB score-severe and 36 as TB score-non-severe. The corresponding exploratory ROC analysis yielded an apparent AUC of 0.8041 (95% CI, 0.6900–0.9182; P = 0.0002). At an exploratory cutoff of 57.80%, sensitivity was 89.47% and specificity was 66.67% (**Supplementary Figure S1C**).

Together, these complementary analyses indicate that the association between the KLRG1^+^CD28^⁻^CD57^+^ Ag85A-specific CD8^+^ T cell phenotype and PTB severity was not dependent solely on the original study-specific symptom-plus-radiological classification. The concordant associations with an established clinical severity score and a published CT-based measure of radiological burden support the robustness of the observed severity-associated pattern. Nevertheless, these analyses remain exploratory and should not be interpreted as external validation of the biomarker or of the original severity classification.

### 3.6 β-galactosidase activity and Ki-67 expression in Ag85A-specific CD8^+^ T cells after short-term stimulation

To further examine the interpretation of β-galactosidase activity after short-term stimulation, additional experiments were performed using PBMCs from three independent patients with PTB (biological n = 3). In addition to senescence-associated β-galactosidase activity, Ki-67 expression was assessed within Ag85A_48_-_56_ tetramer^+^CD8^+^ T cells as a proliferation-associated readout. Polymyxin B was included in the Ag85A-only conditions as an additional control for potential endotoxin-sensitive effects (**Figures 10A–C**).

**Figure 10:**
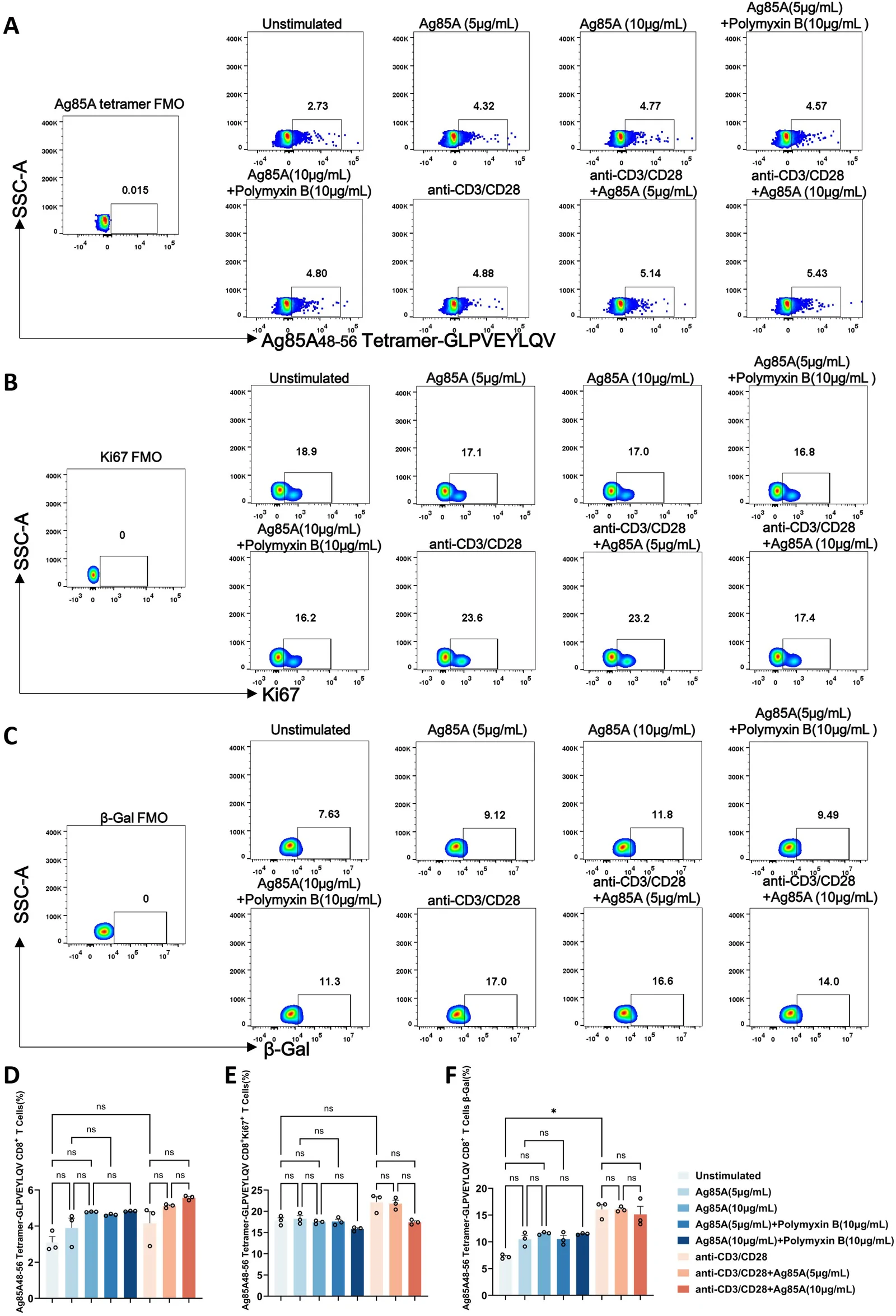
β-galactosidase activity and Ki-67 expression in Ag85A-specific CD8^+^ T cells after 48-h *in vitro* stimulation. PBMCs from three independent patients with PTB (n = 3) were cultured for 48 h under the indicated conditions: unstimulated control, Ag85A at 5μg/mL, Ag85A at 10μg/mL, Ag85A at 5μg/mL plus polymyxin B, Ag85A at 10μg/mL plus polymyxin B, anti-CD3/CD28 stimulation alone, anti-CD3/CD28 plus Ag85A at 5 μg/mL, and anti-CD3/CD28 plus Ag85A at 10μg/mL. Polymyxin B was used at 10μg/mL. **(A)** Representative flow-cytometric plots of Ag85A_48_-_56_ tetramer staining. **(B)** Representative plots of Ki-67 expression within Ag85A_48_-_56_ tetramer^+^CD8^+^ T cells. **(C)** Representative plots of senescence-associated β-galactosidase activity within Ag85A_48_-_56_ tetramer^+^CD8^+^ T cells. **(D–F)** Quantitative comparisons of Ag85A_48_-_56_ tetramer^+^CD8^+^ T cell frequency, Ki-67^+^ cells, and cells with detectable senescence-associated β-galactosidase activity, respectively. Each data point represents one independent PTB donor. Differences across the eight matched stimulation conditions were evaluated using the Friedman test followed by Dunn’s multiple-comparison test for nine prespecified pairwise contrasts. The overall Friedman tests were significant for Ag85A_48_-_56_ tetramer^+^CD8^+^ T cell frequency (P = 0.0058), Ki-67 expression (P = 0.0201), and β-galactosidase activity (P = 0.0087). For panels D and E, none of the nine prespecified pairwise comparisons remained statistically significant after Dunn’s adjustment (all adjusted P > 0.05). For panel F, β-galactosidase activity was significantly higher under anti-CD3/CD28 stimulation than in the unstimulated condition (Dunn-adjusted P = 0.0139). Addition of Ag85A at either 5 or 10 μg/mL to anti-CD3/CD28 stimulation did not significantly alter β-galactosidase activity relative to anti-CD3/CD28 stimulation alone (both Dunn-adjusted P > 0.9999), and Ag85A alone did not differ significantly from the unstimulated condition (Dunn-adjusted P > 0.05). All reported pairwise P values are Dunn-adjusted P values.

Cell viability was directly assessed after the 48-h culture period. Across the indicated stimulation conditions, viability ranged from 76.3% to 99.8%, with the majority of cells remaining viable. Mean viability decreased from 97.2% in the unstimulated condition to 78.7% under anti-CD3/CD28 plus 10μg/mL Ag85A, indicating a descriptive reduction in cell viability under the stronger stimulation condition.

The frequency of Ag85A_48_-_56_ tetramer^+^CD8^+^ T cells showed an overall difference across the eight stimulation conditions (Friedman statistic = 19.89, P = 0.0058). However, none of the nine prespecified pairwise comparisons remained statistically significant after Dunn’s multiple-comparison adjustment (**Figure 10D**). Ki-67 expression similarly showed an overall difference across stimulation conditions (Friedman statistic = 16.60, P = 0.0201), whereas none of the prespecified pairwise comparisons remained significant after Dunn’s adjustment (**Figure 10E**). Dunn-adjusted pairwise P values are provided in the **Figure 10** legend.

Senescence-associated β-galactosidase activity also differed overall across the eight stimulation conditions (Friedman statistic = 18.84, P = 0.0087). Among the nine prespecified pairwise contrasts, β-galactosidase activity was significantly higher under anti-CD3/CD28 stimulation than in the unstimulated condition after Dunn’s multiple-comparison adjustment. In contrast, addition of Ag85A to anti-CD3/CD28 stimulation did not significantly alter β-galactosidase activity compared with anti-CD3/CD28 stimulation alone, and Ag85A alone did not differ significantly from the unstimulated condition (**Figure 10F**). Dunn-adjusted pairwise P values are provided in the **Figure 10** legend.

Together, these findings indicate overall condition-associated variation in Ag85A_48_-_56_ tetramer^+^CD8^+^ T cell frequency, Ki-67 expression, and β-galactosidase activity. However, the only prespecified pairwise difference that remained significant after correction for multiple testing was the increase in β-galactosidase activity under anti-CD3/CD28 stimulation relative to the unstimulated condition. These findings therefore do not support a specific Ag85A-dependent increase in β-galactosidase activity. Given the small number of independent donors, these observations should be considered exploratory.

Importantly, increased β-galactosidase activity after 48 h of culture may accompany activation-associated changes in lysosomal activity. Accordingly, the β-galactosidase findings were interpreted as changes in a senescence-associated lysosomal readout rather than as direct evidence of irreversible T-cell senescence.

### 3.7 Intracellular IFN-γ and TNF-α responses in Ag85A-specific CD8^+^ T cells under defined *in vitro* stimulation conditions

To evaluate intracellular cytokine responses without additional PMA/ionomycin-mediated polyclonal restimulation of the Ag85A-treated cells, the IFN-γ and TNF-α experiments were repeated using a revised stimulation protocol. PBMCs from three independent patients with PTB were cultured under six conditions: unstimulated control, PMA/ionomycin positive control, Ag85A at 5μg/mL, Ag85A at 10μg/mL, Ag85A at 5μg/mL plus polymyxin B (10μg/mL), and Ag85A at 10μg/mL plus polymyxin B (10μg/mL) (**Figures 3A**, **4A**). Brefeldin A and monensin were added to all conditions during the final 4 h of culture to permit intracellular cytokine accumulation, whereas PMA/ionomycin was restricted to the separate positive-control condition.

Ag85A_48_-_56_ tetramer^+^CD8^+^ T cells remained detectable across all experimental conditions, and CD28/CD57 expression within the KLRG1^+^ Ag85A_48_-_56_ tetramer^+^CD8^+^ T cell population was evaluated under the same conditions (**Figures 3B**, **4B**). Intracellular IFN-γ and TNF-α signals were quantified as mean fluorescence intensity (MFI) within KLRG1^+^CD28^⁻^CD57^+^ Ag85A-specific CD8^+^ T cells (**Figures 3C**, **4C**).

Intracellular IFN-γ MFI differed significantly overall across the six matched stimulation conditions (Friedman statistic = 14.62, exact P < 0.0001). Dunn-adjusted prespecified pairwise comparisons showed that the PMA/ionomycin positive-control condition had significantly higher IFN-γ MFI than the unstimulated condition. Although mean IFN-γ MFI was numerically higher following Ag85A stimulation at 5 μg/mL than in the unstimulated condition, this difference did not remain statistically significant after multiplicity adjustment. No significant differences were observed between the unstimulated and Ag85A 10μg/mL conditions, between the corresponding Ag85A-only and polymyxin B-containing conditions, or between the 5 and 10μg/mL Ag85A conditions. Dunn-adjusted pairwise P values are provided in the **Figure 3** legend (**Figure 3C**).

Intracellular TNF-α MFI also differed significantly overall across the six matched stimulation conditions (Friedman statistic = 14.62, exact P < 0.0001). Dunn-adjusted prespecified pairwise comparisons showed that the PMA/ionomycin positive-control condition had significantly higher TNF-α MFI than the unstimulated condition. Although mean TNF-α MFI was numerically higher following Ag85A stimulation at 5 μg/mL than in the unstimulated condition, this difference did not remain statistically significant after multiplicity adjustment. No significant differences were observed between the unstimulated and Ag85A 10μg/mL conditions, between the corresponding Ag85A-only and polymyxin B-containing conditions, or between the 5 and 10μg/mL Ag85A conditions. Dunn-adjusted pairwise P values are provided in the **Figure 4** legend (**Figure 4C**).

Addition of polymyxin B therefore did not produce a statistically significant alteration in either IFN-γ or TNF-α MFI relative to the corresponding Ag85A-only conditions. Because the Ag85A-containing groups were not subjected to additional PMA/ionomycin stimulation, the cytokine signals measured in these groups represent intracellular signals observed under the indicated Ag85A culture conditions rather than responses elicited by subsequent PMA/ionomycin-mediated polyclonal restimulation. However, none of the Ag85A-containing conditions differed significantly from the unstimulated condition after multiplicity adjustment. Accordingly, these findings should be interpreted as exploratory measurements of intracellular cytokine responses under Ag85A-containing culture conditions rather than evidence that Ag85A significantly induces IFN-γ or TNF-α production. The significant response to the PMA/ionomycin positive control nevertheless confirmed the capacity of the analyzed cells to mount detectable intracellular cytokine responses. Given the small number of independent PTB donors and the cellular complexity of PBMC cultures, these findings do not establish a direct Ag85A-driven cytokine-production mechanism.

## 4 Discussion

The occurrence and clinical severity of PTB reflect a prolonged and dynamic interaction between Mtb and the host immune system (Chandra et al., 2022; Trajman et al., 2025). As a chronic granulomatous disease, PTB is characterized by persistent or recurrent antigen exposure, tissue inflammation, and heterogeneous bacillary burden (Ramakrishnan, 2012; Kaufmann and Dorhoi, 2013; Chandra et al., 2022; Trajman et al., 2025). In this clinical context, antigen-specific T-cell populations may undergo progressive differentiation and acquire highly differentiated phenotypes, some of which have also been associated with immunosenescence (Brenchley et al., 2003; McShane et al., 2004; Goronzy and Weyand, 2019; Liu et al., 2023; Terekhova et al., 2025). In the present study, we found that KLRG1^+^CD28^⁻^CD57^+^ Ag85A-specific CD8^+^ T cells were increased in patients with PTB and were associated with sputum smear positivity, higher smear grade, severe disease, lower lymphocyte percentage, and exploratory treatment-related outcome groups. These findings support the relevance of KLRG1^+^CD28^⁻^CD57^+^ Ag85A-specific CD8^+^ T cells as an antigen-specific immune correlate of PTB activity and severity. Taken together, our results suggest that this phenotype may represent an antigen-specific terminal-differentiation imprint of active and severe PTB. Rather than reflecting generalized immune aging alone, this phenotype appears to map onto a clinically meaningful continuum that includes active disease, higher smear-defined bacillary burden, lower lymphocyte percentage, and severe pulmonary involvement. This interpretation places Ag85A-specific CD8^+^ T cell differentiation at the interface between Mtb-associated antigenic stimulation and host disease phenotype.

This study focused on Ag85A-specific CD8^+^ T cells because Ag85A is an immunodominant antigen within the antigen 85 complex and has been widely studied in Mtb-specific T-cell immunity (Huygen, 2014). Human Ag85A-specific CD8^+^ cytotoxic T-cell responses, including HLA-A*02:01-restricted responses to Ag85A-derived epitopes, have been previously reported (Smith et al., 2000). In addition, Mtb-specific CD8^+^ T cells have been shown to differ phenotypically and functionally between LTBI and active TB, supporting the relevance of antigen-specific CD8^+^ T cell profiling in human tuberculosis (Rozot et al., 2013). In the present cohort, patients with PTB showed higher frequencies of Ag85A-specific CD8^+^ T cells than HCs across all age strata. This finding is compatible with the interpretation that active PTB is accompanied by increased peripheral detection or enrichment of Ag85A-specific CD8^+^ T cells. However, this should not be interpreted as evidence that detectable Ag85A-specific CD8^+^ T cells are exclusively generated by active disease, because prior BCG vaccination and environmental mycobacterial exposure may contribute to baseline Ag85A-related memory responses, particularly in HCs (McShane et al., 2004; Huygen, 2014).

A central observation of this study was the increased proportion of KLRG1^+^CD28^⁻^CD57^+^ Ag85A-specific CD8^+^ T cells in patients with PTB. CD28 loss, KLRG1 expression, and CD57 expression are commonly associated with advanced T-cell differentiation, reduced proliferative potential, and immunosenescence-associated phenotypes (Voehringer et al., 2002; Brenchley et al., 2003; Goronzy and Weyand, 2019; Liu et al., 2023; Terekhova et al., 2025). Therefore, the KLRG1^+^CD28^⁻^CD57^+^ phenotype was used here operationally to identify Ag85A-specific CD8^+^ T cells with terminal differentiation- and immunosenescence-associated features. Importantly, the present data do not establish that persistent Ag85A exposure alone causes this phenotype *in vivo*. Rather, they indicate that KLRG1^+^CD28^⁻^CD57^+^ Ag85A-specific CD8^+^ T cells are closely associated with active PTB and are more prominent in clinically severe disease. This interpretation is consistent with an observational translational study design and avoids overstating mechanistic causality.

The association between KLRG1^+^CD28^⁻^CD57^+^ Ag85A-specific CD8^+^ T cells and sputum smear status further supports a relationship between this phenotype and smear-defined clinical disease burden. Smear-positive patients had higher proportions of KLRG1^+^CD28^⁻^CD57^+^ Ag85A-specific CD8^+^ T cells than smear-negative patients, and patients with higher smear grades also exhibited higher proportions of this subset. Because sputum smear grade is a clinical surrogate of bacillary burden rather than a direct quantitative measure of viable Mtb load, these findings suggest that the KLRG1^+^CD28^⁻^CD57^+^ phenotype coexists with higher smear-defined bacillary burden. In the exploratory follow-up analysis, patients who achieved sputum smear conversion after 2 months showed lower baseline proportions of KLRG1^+^CD28^⁻^CD57^+^ Ag85A-specific CD8^+^ T cells than those who remained smear-positive after 6 months. However, this comparison involved small follow-up groups, immunophenotyping was performed only at baseline, and the follow-up categories represented heterogeneous clinical states rather than prospectively defined prognostic endpoints. The death category was additionally based on all-cause mortality. Accordingly, these observations are descriptive and hypothesis-generating only and do not establish that this phenotype predicts sputum conversion, treatment failure, mortality, or other clinical outcomes. Larger prospectively designed longitudinal studies with predefined endpoints are required before any prognostic interpretation can be made.

The exploratory associations between KLRG1^+^CD28^⁻^CD57^+^ Ag85A-specific CD8^+^ T cells and baseline host-status parameters provide additional context for this severity-associated immune phenotype. Before correction for multiple testing, nominal associations were observed with neutrophil percentage, lymphocyte percentage, CRP, and albumin. After Benjamini–Hochberg FDR correction across the 12 correlation analyses, higher neutrophil percentage, lower lymphocyte percentage, and higher CRP remained significantly associated with the phenotype, whereas the inverse association with albumin did not remain significant (**Figure 5**). These findings indicate that enrichment of this phenotype accompanies selected hematologic and inflammatory features of disease burden, although these cross-sectional correlations do not establish causality or independence from overall disease severity.

The plasma cytokine results should also be interpreted cautiously. After accounting for their skewed distributions using Spearman rank correlation, neither TNF-α nor IFN-γ showed a statistically significant association with the antigen-specific phenotype. Plasma TNF-α also did not differ significantly between severe and non-severe PTB and was numerically lower in the severe group, whereas IFN-γ was numerically higher in severe PTB but without a statistically significant between-group difference (**Supplementary Table S2**). Taken together, these findings do not provide robust evidence that the antigen-specific terminal-differentiation phenotype co-segregates with a specific systemic TNF-α- or IFN-γ-associated inflammatory signature.

Plasma cytokine measurements and intracellular cytokine responses within Ag85A-specific CD8^+^ T cells represent biologically distinct readouts and should not be considered directly equivalent. Plasma cytokine concentrations reflect the integrated systemic immune response arising from multiple immune-cell and tissue compartments during tuberculosis (Kaufmann and Dorhoi, 2013; Chandra et al., 2022). In contrast, Ag85A_48_-_56_ tetramer^+^CD8^+^ T cells represent a defined antigen-specific CD8^+^ T cell population. Therefore, the present plasma cytokine measurements should be interpreted independently from the antigen-specific terminal-differentiation phenotype rather than as direct evidence of altered cytokine production by the KLRG1^+^CD28^⁻^CD57^+^ Ag85A-specific CD8^+^ T cell subset or of a shared inflammatory mechanism.

The *in vitro* stimulation experiments provide additional but exploratory context for interpretation of the β-galactosidase readout. Overall condition-associated variation was detected in Ag85A_48_-_56_ tetramer^+^CD8^+^ T cell frequency, Ki-67 expression, and β-galactosidase activity. However, most prespecified pairwise comparisons did not remain significant after correction for multiple testing. Notably, β-galactosidase activity was significantly higher following anti-CD3/CD28 stimulation than in the unstimulated condition, whereas addition of Ag85A at either concentration did not produce a further significant increase relative to anti-CD3/CD28 stimulation alone. Similarly, no specific pairwise difference in Ki-67 expression remained significant after multiplicity adjustment. This pattern suggests that increased β-galactosidase activity in short-term stimulated T cells may partly reflect activation-associated lysosomal changes rather than an Ag85A-specific induction of irreversible cellular senescence. In addition, viability was lower under stronger stimulation conditions, indicating that cell survival should also be considered when interpreting these short-term responses. Accordingly, β-galactosidase activity in this study is interpreted as a senescence-associated lysosomal readout rather than definitive evidence of T cell senescence.

The revised intracellular cytokine experiments were designed to address an important limitation of the original stimulation protocol. Brefeldin A and monensin were applied uniformly across all culture conditions during the final 4 h to permit intracellular cytokine accumulation, whereas PMA/ionomycin was restricted to a separate positive-control condition. Significant overall variation in both IFN-γ and TNF-α MFI was observed across the stimulation conditions. However, after correction for multiple testing, only the PMA/ionomycin positive-control condition differed significantly from the unstimulated condition. Ag85A at 5μg/mL produced numerically higher mean IFN-γ and TNF-α signals than the unstimulated condition, but neither comparison remained statistically significant after multiplicity adjustment. Likewise, polymyxin B did not significantly alter the cytokine signals relative to the corresponding Ag85A-only conditions. These findings confirm that the analyzed cells were capable of mounting detectable intracellular cytokine responses under strong polyclonal stimulation, but they do not provide statistically robust evidence that Ag85A itself significantly increased IFN-γ or TNF-α production in this small exploratory experiment. Because PBMC cultures contain multiple immune-cell populations and antigen-presenting cells, and because only three independent PTB donors were examined, the observed cytokine patterns should be interpreted cautiously as exploratory intracellular responses under the indicated culture conditions rather than as evidence of a direct Ag85A-driven cytokine-production mechanism.

Another clinically relevant finding was the consistent association between the KLRG1^+^CD28^⁻^CD57^+^ Ag85A-specific CD8^+^ T cell phenotype and PTB severity. After accounting for unequal variances using Welch’s ANOVA followed by Dunnett’s T3-adjusted comparisons, patients with severe PTB had significantly higher proportions of this subset than patients with non-severe PTB, individuals with LTBI, and HCs. Patients with non-severe PTB also showed higher proportions than HCs, whereas no significant difference was observed between non-severe PTB and LTBI. These findings indicate that enrichment of this terminal differentiation-associated phenotype was most pronounced in clinically severe PTB rather than reflecting a simple stepwise increase across all infection states. In the exploratory multivariable logistic regression model, the proportion of KLRG1^+^CD28^⁻^CD57^+^ Ag85A-specific CD8^+^ T cells remained associated with severe PTB after adjustment for variables significant in univariable analysis. Collectively, these observations suggest that this antigen-specific terminal differentiation-associated phenotype may reflect clinically meaningful immune remodeling associated with more severe active TB. However, the directionality of this relationship cannot be determined from the current observational data. Greater disease burden, systemic inflammation, tissue pathology, and altered host immunity may contribute to the accumulation of this phenotype, while the phenotype itself may primarily mark an immune state associated with advanced disease rather than directly contributing to disease progression.

Consistent with this severity-associated pattern, ROC analysis showed exploratory in-cohort discrimination between severe and non-severe PTB, with an apparent AUC of 0.8333 and a bootstrap bias-corrected AUC of 0.8333. The severity-associated pattern was also supported by positive correlations with the Bandim TB score and the CT-based radiological burden score. When clinical severity was alternatively defined using the established TB score threshold of≥8 versus <8, the phenotype retained exploratory discriminative ability, with an apparent AUC of 0.8041 and a bootstrap bias-corrected AUC of 0.8039. These complementary findings support the internal robustness of the severity-associated pattern across alternative severity measures but do not constitute external validation.

Bootstrap resampling evaluates internal stability but does not establish generalizability to independent populations. Accordingly, all ROC analyses remain exploratory, and independent external validation in larger, prospectively recruited cohorts is required before the observed AUCs or exploratory cutoff values can be considered for clinical severity stratification (Steyerberg and Harrell, 2016).

This study has several limitations. First, the primary comparisons among HCs, individuals with LTBI, and patients with PTB, including severity-stratified PTB subgroups, were cross-sectional. Therefore, the observed associations between KLRG1^+^CD28^⁻^CD57^+^ Ag85A-specific CD8^+^ T cells and disease severity do not establish temporal or causal relationships. Although exploratory longitudinal follow-up was conducted in sputum smear-positive patients with PTB, immunophenotyping was performed only at baseline, and the number of patients within each exploratory outcome group was limited. Consequently, the follow-up findings should be interpreted as preliminary associations rather than evidence of validated prognostic performance.

Second, this was a single-center study without an independent external validation cohort. Center-specific factors, including patient characteristics, local clinical management, laboratory workflows, BCG vaccination background, and regional population characteristics, may have influenced the findings. This limitation is particularly relevant to the ROC analysis. The AUC of 0.8333 and the exploratory cutoff value of 57.80% reflect in-cohort discriminative performance only and may overestimate performance in external populations. Prospective multicenter studies with independent validation cohorts are needed to evaluate the robustness and reproducibility of the observed association between this antigen-specific phenotype and PTB severity (Steyerberg and Harrell, 2016).

Third, universal neonatal BCG vaccination in China represents an important background confounder. Because BCG contains antigenic components related to the Ag85 complex and Ag85A-based vaccine platforms have been shown to boost BCG-primed and naturally acquired antimycobacterial immunity in humans, baseline Ag85A-related memory responses in HCs are expected (McShane et al., 2004; Huygen, 2014). Therefore, detectable Ag85A-specific CD8^+^ T-cell populations in HCs should not be interpreted as unexpected or necessarily pathological. Instead, they likely reflect a combination of BCG-elicited memory and population-level mycobacterial exposure. This background may reduce contrast between HCs and infected groups and should be carefully considered when interpreting Ag85A-specific immune responses.

Fourth, an important limitation of the present study is that the Ag85A_48_–_56_ tetramer is HLA-A*02:01-restricted (Smith et al., 2000), whereas formal HLA genotyping was not performed. Therefore, detection of a tetramer-positive CD8^+^ T-cell population cannot be considered equivalent to confirmation of HLA-A*02:01 genotype. Conversely, absent or insufficient tetramer staining may reflect HLA-A*02:01 mismatch, a genuinely low frequency of Ag85A-reactive CD8^+^ T cells, or technical factors. Because these possibilities could not be distinguished, restricting downstream analyses to participants with a discernible tetramer-positive population may have introduced selection bias and may have enriched the analyzed cohort for individuals with more readily detectable tetramer-reactive CD8^+^ T-cell populations. Accordingly, the between-group comparisons reported here should be interpreted as applying to this analyzable tetramer-positive subset rather than to the entire PTB, LTBI, or HC populations. Future studies incorporating prospective HLA-A*02:01 typing, predefined tetramer-positive event thresholds, and appropriate tetramer-specific controls will be important to validate and generalize these findings.

Fifth, age-dependent confounding should be considered. Although participants were analyzed using predefined age strata, residual age-related confounding across HCs, individuals with LTBI, and patients with PTB may still have influenced the observed immune phenotypes. This is particularly relevant because thymic output, memory T-cell accumulation, and immunosenescence-associated phenotypes vary with age (Nikolich-Žugich, 2018; Goronzy and Weyand, 2019; Deng et al., 2025). The LTBI cohort may also differ from the PTB and HC groups in unmeasured exposure history, estimated infection duration, prior mycobacterial exposure, and host immune background. Larger age-matched and prospectively recruited cohorts are needed to confirm whether the observed associations remain stable across age groups.

Sixth, the study remains primarily phenotypic and observational. Without mechanistic interventions such as receptor blockade, pathway-specific inhibition, antigen-presentation manipulation, or rescue experiments, we cannot determine whether the KLRG1^+^CD28^⁻^CD57^+^ phenotype reflects immunosenescence, exhaustion-like differentiation, chronic activation, or overlapping differentiation and dysfunction states. Thus, the current data support a clinically relevant antigen-specific immune association but do not identify a causal molecular pathway.

Seventh, the *in vitro* stimulation experiments used a short-term 48-h acute ex vivo culture model. Senescence-associated β-galactosidase activity alone does not establish irreversible cellular senescence, particularly in short-term stimulated lymphocytes, because increased lysosomal β-galactosidase activity may also accompany cellular activation. In the additional experiments, Ki-67 was therefore assessed together with β-galactosidase activity, and polymyxin B was included in selected Ag85A conditions to evaluate potential endotoxin-sensitive effects. Nevertheless, these experiments involved only three independent PTB donors and should therefore be regarded as exploratory. Moreover, most prespecified pairwise comparisons in the β-galactosidase/Ki-67 experiments did not remain significant after multiplicity adjustment, while the viability measurements were used only as descriptive quality-control data. These considerations further limit the strength of conclusions regarding specific stimulation-associated effects. The 48-h culture system also cannot reproduce the chronic, compartmentalized, and dynamic antigen exposure occurring during human PTB. Accordingly, these findings should be interpreted as short-term stimulation-associated cellular changes rather than direct proof of antigen-driven T-cell senescence *in vivo*.

Finally, residual confounding remains possible. Given the limited size of the PTB cohort and the number of severe disease events, the primary multivariable model was deliberately parsimonious and did not simultaneously include all potentially relevant clinical covariates. Age may influence T cell differentiation independently of tuberculosis severity, whereas sputum smear status may reflect bacillary burden and may therefore be closely related to the disease-severity pathway. Diabetes mellitus may alter host immune responses and systemic inflammatory status, while respiratory failure may represent a manifestation or downstream consequence of severe pulmonary disease rather than an independent baseline confounder. Similarly, inflammatory indices may both correlate with the immune phenotype and constitute components of the biological pathway linking disease burden to clinical severity. Treatment status and treatment timing may also influence immune activation and T cell differentiation. Accordingly, residual effects of age, smear-defined bacillary burden, diabetes, respiratory failure, systemic inflammatory status, and treatment status cannot be excluded.

Other incompletely captured factors, including smoking history, duration of symptoms before enrollment, prior subclinical mycobacterial exposure, glycemic control, broader nutritional status beyond the anthropometric variables incorporated into TB score and serum albumin, and chronic inflammatory conditions, may also have influenced the observed associations.

In conclusion, patients with PTB exhibited increased proportions of KLRG1^+^CD28^⁻^CD57^+^ Ag85A-specific CD8^+^ T cells, and this phenotype was associated with sputum smear status, higher smear grade, lower lymphocyte percentage, and disease severity. Exploratory ROC analysis suggested in-cohort discriminative potential for distinguishing severe from non-severe PTB, but the AUC and cutoff value require external validation before clinical application. Overall, these findings support KLRG1^+^CD28^⁻^CD57^+^ Ag85A-specific CD8^+^ T cells as a candidate translational correlate of PTB severity rather than a proven mechanistic driver or validated biomarker. Larger, prospectively recruited, HLA-genotyped, age-matched, multicenter cohorts with longitudinal follow-up and functional or mechanistic validation are required to confirm and extend these observations.

## Data Availability

The raw data supporting the conclusions of this article will be made available by the authors, without undue reservation.

## Supplementary material

Supplementary Figure 1 Associations of KLRG1^+^CD28^⁻^CD57^+^ Ag85A-specific CD8^+^ T cells with alternative measures of pulmonary tuberculosis severity and corresponding exploratory ROC analyses. **(A)** Representative chest CT images illustrating radiological differences between patients with non-severe and severe pulmonary tuberculosis (PTB). Three representative cases are shown for each severity category. **(B)** Spearman correlation between the proportion of KLRG1^+^CD28^⁻^CD57^+^ cells among Ag85A_48_-_56_ tetramer^+^CD8^+^ T cells and TB score. The proportion of this subset was positively correlated with TB score (Spearman ρ = 0.5662, P <0.0001; n = 55). **(C)** Exploratory ROC analysis evaluating the in-cohort discriminative potential of the KLRG1^+^CD28^⁻^CD57^+^ Ag85A-specific CD8^+^ T-cell proportion for distinguishing TB score-defined severe PTB (TB score ≥8; n = 19) from TB score-defined non-severe PTB (TB score <8; n = 36). The apparent AUC was 0.8041 (95% CI, 0.6900–0.9182; P = 0.0002). At an exploratory cutoff of 57.80%, sensitivity was 89.47% and specificity was 66.67%. Internal bootstrap results are provided in **Supplementary Table S3**. **(D)** Spearman correlation between the proportion of KLRG1^+^CD28^⁻^CD57^+^ cells among Ag85A_48_-_56_ tetramer^+^CD8^+^ T cells and CT score in 52 patients with available CT score data. The phenotype was positively correlated with CT score (Spearman ρ = 0.5413, P < 0.0001; n = 52).
