# Supplementary material for: KLRG1+CD28⁻CD57+ Ag85A-specific CD8+ T cells mark a severity-associated terminal-differentiation imprint in pulmonary tuberculosis
Source: Front Cell Infect Microbiol. 2026 Oct 1;16:1938907. doi: 10.3389/fcimb.2026.1938907 (PMC13668559; doi:10.3389/fcimb.2026.1938907)
Supplement: Supplementary file 1 [file Table1.docx]

| **Supplementary Table S1.** Demographic, Clinical, and Laboratory Characteristics of Participants in the PTB, LTBI, and HC Groups. | | | | | |
| --- | --- | --- | --- | --- | --- |
| **Characteristics** |  | **PTB Group** | **LTBI Group** | **HC Group** | **P Value** |
| **Number of participants, n** |  | 55 | 33 | 36 | - |
| **Gender, n (%)** | Male | 30 (54.5%) | 16 (48.5%) | 18 (50%) | 0.670 |
|  | Female | 25 (45.5%) | 17 (51.5%) | 18 (50%) |  |
| **Age (years)** |  | 34 (24, 52) | 32.5 (14, 47.5) | 31.5 (14, 55) | 0.408 |
| **Disease Severity, n (%)** | Non-severe | 30 (54.5%) | - | - | - |
|  | Severe | 25 (45.5%) | - | - | - |
| **Etiological Confirmation, n (%)** |  |  |  |  | - |
| Sputum smear for acid-fast bacilli, Positive |  | 30 (54.5%) | - | - | - |
| Sputum Xpert MTB/RIF or TB-DNA, Positive |  | 17 (30.9%) | - | - | - |
| **Radiological Findings, n (%)** |  |  |  |  | - |
| Lesions involving ≥3 lung fields |  | 21 (38.2%) | - | - | - |
| Cavitation |  | 9 (16.4%) | - | - | - |
| **Clinical Symptoms, n (%)** |  |  |  |  | - |
| Cough |  | 38 (69.1%) | - | - | - |
| Expectoration |  | 35 (63.6%) | - | - | - |
| Hemoptysis |  | 2 (3.6%) | - | - | - |
| Chest pain |  | 2 (3.6%) | - | - | - |
| Dyspnea |  | 6 (10.9%) | - | - | - |
| Fatigue |  | 10 (18.2%) | - | - | - |
| Anorexia |  | 3 (5.5%) | - | - | - |
| Weight loss |  | 5 (9.1%) | - | - | - |
| Fever |  | 8 (14.5%) | - | - | - |
| Night sweats |  | 5 (9.1%) | - | - | - |
| **Comorbidities, n (%)** |  |  |  |  | - |
| Bronchial tuberculosis |  | 15 (27.3%) | - | - | - |
| Tuberculous pleurisy / Poly-serositis |  | 8 (14.5%) | - | - | - |
| Extrapulmonary tuberculosis |  | 6 (10.9%) | - | - | - |
| Hypertension |  | 6 (10.9%) | - | - | - |
| Diabetes mellitus |  | 8 (14.5%) | - | - | - |
| COPD |  | 1 (1.8%) | - | - | - |
| Respiratory failure |  | 6 (10.9%) | - | - | - |
| **Laboratory Parameters** |  |  |  |  |  |
| White blood cell count (×10⁹/L) |  | 7.49 (6.36, 8.63) | 5.80 (5.33, 6.27) | 6.18 (5.76, 6.60) | - |
| Hemoglobin (g/L) |  | 118.05 (112.34, 123.77) | 139.27 (134.74, 143.80) | 140.39 (134.64, 146.14) | - |
| Neutrophil percentage (%) |  | 66.68 (62.90, 70.47) | 54.06 (51.11, 57.01) | 55.04 (52.16, 57.93) | - |
| Lymphocyte percentage (%) |  | 21.91 (18.68, 25.14) | 34.77 (31.73, 37.81) | 35.08 (32.30, 37.86) | - |
| Monocyte percentage (%) |  | 9.23 (6.01, 12.45) | 7.89 (7.29, 8.49) | 7.11 (6.69, 7.52) | - |
| C-reactive protein (mg/L) |  | 14.10 (0.50, 44.65) | - | - | - |
| Albumin (g/L) |  | 37.50 (32.50, 41.30) | - | - | - |
| Globulin (g/L) |  | 28.40 (25.10, 35.80) | - | - | - |
| Albumin-to-globulin ratio (A/G) |  | 1.30 (1.10, 1.60) | - | - | - |
| IL-5 (pg/L) |  | 2.65 (2.23, 3.60) | - | - | - |
| IFN-α (pg/L) |  | 1.80 (1.32, 2.03) | - | - | - |
| IL-2 (pg/L) |  | 2.09 (1.84, 2.31) | - | - | - |
| IL-6 (pg/L) |  | 4.82 (3.18, 9.44) | - | - | - |
| IL-1β (pg/L) |  | 3.79 (2.27, 8.90) | - | - | - |
| IL-10 (pg/L) |  | 1.72 (1.45, 2.00) | - | - | - |
| IFN-γ (pg/L) |  | 6.75 (3.39, 11.90) | - | - | - |
| IL-8 (pg/L) |  | 7.20 (2.28, 14.84) | - | - | - |
| IL-17 (pg/L) |  | 2.27 (2.03, 2.79) | - | - | - |
| IL-4 (pg/L) |  | 1.72 (1.58, 1.88) | - | - | - |
| IL-12P70 (pg/L) |  | 1.70 (1.64, 1.84) | - | - | - |
| TNF-α (pg/L) |  | 2.36 (1.72, 3.27) | - | - | - |
| **History of TB contact, n (%)** | Yes | 13 (23.6%) | 33 (100%) | 0 (0%) | < 0.001 |
|  | No | 42 (76.4%) | 0 (0%) | 36 (100%) | - |
| **Length of hospital stay (days), n (%)** | ＜7 | 6 (10.9%) |  |  | < 0.01 |
|  | 7-15 | 24 (43.6%) |  |  |  |
|  | 16-30 | 15 (27.3%) |  |  |  |
|  | ＞30 | 10 (18.2%) |  |  |  |

Supplementary Table S1 Clinical characteristics, laboratory findings, and chest CT features of patients with pulmonary tuberculosis, individuals with latent tuberculosis infection, and healthy controls.
