# Supplementary material for: KLRG1+CD28⁻CD57+ Ag85A-specific CD8+ T cells mark a severity-associated terminal-differentiation imprint in pulmonary tuberculosis
Source: Front Cell Infect Microbiol. 2026 Oct 1;16:1938907. doi: 10.3389/fcimb.2026.1938907 (PMC13668559; doi:10.3389/fcimb.2026.1938907)
Supplement: Supplementary file 2 [file Table2.docx]

| **Supplementary Table S2.** Comparison of clinical, radiological, and laboratory characteristics between non-severe and severe PTB groups. | | | |
| --- | --- | --- | --- |
| **Characteristics** | **Non-severe PTB Group (n=30)** | **Severe PTB Group (n=25)** | **Nominal**  **P value** |
| **Immunophenotypic variable** |  |  |  |
| Ag85A48-56 Tetramer  KLRG1^+^CD28^-^CD57^+^CD8^+^ T Cells | 48.91±13.39 | 66.93±12.23 | < 0.001 |
| **Clinical Symptoms and Radiological Findings, n (%)** |  |  |  |
| Involved lung fields | 2.00 (1.00, 2.00) | 4.00 (4.00, 4.00) | < 0.001 |
| Cavitation | 0 (0.0%) | 9 (36.0%) | < 0.001 |
| Cough | 16 (53.3%) | 22 (88.0%) | 0.008 |
| Expectoration | 14 (46.7%) | 21 (84.0%) | 0.005 |
| Dyspnea | 0 (0.0%) | 6 (24.0%) | 0.004 |
| Fatigue | 1 (3.3%) | 9 (36.0%) | < 0.001 |
| Anorexia | 2 (6.7%) | 1 (4.0%) | 1.000 |
| Chest pain | 1 (3.3%) | 1 (4.0%) | 1.000 |
| Hemoptysis | 0 (0.0%) | 2 (8.0%) | 0.165 |
| Fever | 3 (10.0%) | 5 (20.0%) | 0.249 |
| Night sweats | 1 (3.3%) | 4 (16.0%) | 0.146 |
| Weight loss | 2 (6.7%) | 3 (12.0%) | 0.637 |
| Comorbidities, n (%) |  |  |  |
| Hypertension | 2 (6.7%) | 4 (16.0%) | 0.382 |
| Diabetes mellitus | 2 (6.7%) | 6 (24.0%) | 0.058 |
| COPD | 0 (0.0%) | 1 (4.0%) | 0.423 |
| Respiratory failure | 0 (0.0%) | 6 (24.0%) | 0.005 |
| Bronchial tuberculosis | 7 (23.3%) | 8 (32.0%) | 0.362 |
| Tuberculous pleurisy / Poly-serositis | 6 (20.0%) | 3 (12.0%) | 0.717 |
| Extrapulmonary tuberculosis | 3 (10.0%) | 3 (12.0%) | 0.689 |
| **Laboratory Parameters** |  |  |  |
| White blood cell count (×10⁹/L) | 5.77 (4.67, 7.29) | 6.45 (5.57, 8.12) | 0.120 |
| Hemoglobin (g/L) | 124.10±14.19 | 109.96±25.49 | 0.012 |
| Neutrophil percentage (%) | 60.10 (56.1, 69.4) | 69.80 (63.5, 75.6) | 0.02 |
| Lymphocyte percentage (%) | 26.50±10.80 | 17.18±10.23 | 0.002 |
| Monocyte percentage (%) | 8.55 (7.33, 10.90) | 9.40 (7.10, 11.60) | 0.510 |
| C-reactive protein (mg/L) | 1.60 (0.50, 11.45) | 14.30 (2.30, 31.92) | 0.044 |
| Albumin (g/L) | 39.30±4.35 | 34.61±7.38 | 0.005 |
| Globulin (g/L) | 29.90 ± 5.54 | 30.92 ± 9.31 | 0.618 |
| Albumin-to-globulin ratio (A/G) | 1.36 ± 0.29 | 1.23 ± 0.47 | 0.229 |
| IL-5 (pg/L) | 2.83±1.04 | 2.60±0.82 | 0.429 |
| IFN-α (pg/L) | 1.86 (1.56, 2.02) | 1.54 (1.32, 1.85) | 0.11 |
| IL-2 (pg/L) | 2.02 (1.89, 2.27) | 2.09 (1.93, 2.47) | 0.702 |
| IL-6 (pg/L) | 4.71 (3.09, 6.82) | 5.70 (3.23, 11.71) | 0.191 |
| IL-1β (pg/L) | 3.86 (2.33, 9.22) | 3.85 (2.29, 7.57) | 0.862 |
| IL-10 (pg/L) | 1.73 (1.53, 2.00) | 1.74 (1.54, 2.61) | 0.853 |
| IFN-γ (pg/L) | 6.57 (3.90, 8.65) | 12.00 (4.24, 26.36) | 0.102 |
| IL-8 (pg/L) | 5.26 (2.38, 12.76) | 9.37 (2.28, 42.81) | 0.256 |
| IL-17 (pg/L) | 2.30 (2.09, 2.61) | 2.42 (2.04, 3.21) | 0.411 |
| IL-4 (pg/L) | 1.71±0.14 | 1.67±0.26 | 0.485 |
| IL-12P70 (pg/L) | 1.70 (1.64, 1.79) | 1.66 (1.57, 1.77) | 0.539 |
| TNF-α (pg/L) | 2.36 (1.85, 2.48) | 1.89 (1.59, 3.42) | 0.643 |

Supplementary Table S2 Baseline clinical characteristics, laboratory parameters, and chest CT findings in patients with severe versus non-severe pulmonary tuberculosis. These comparisons were exploratory and were performed primarily to descriptively characterize the two severity groups. Reported P values are nominal and were not adjusted for multiple comparisons. Given the number of variables examined, nominal P values—particularly those close to 0.05—should be interpreted as hypothesis-generating rather than confirmatory evidence of independent associations with disease severity.
