# Supplementary material for: KLRG1+CD28⁻CD57+ Ag85A-specific CD8+ T cells mark a severity-associated terminal-differentiation imprint in pulmonary tuberculosis
Source: Front Cell Infect Microbiol. 2026 Oct 1;16:1938907. doi: 10.3389/fcimb.2026.1938907 (PMC13668559; doi:10.3389/fcimb.2026.1938907)
Supplement: Supplementary file 3 [file Table3.docx]

**Supplementary Table S3.** Internal bootstrap assessment of exploratory ROC analyses for PTB severity using the KLRG1⁺CD28⁻CD57⁺ Ag85A-specific CD8⁺ T cell phenotype.

| **Outcome definition** | **n high** | **n low** | **Apparent AUC** | **Bootstrap mean AUC** | **Bootstrap median AUC** | **Bootstrap 95% CI** | **Bootstrap bias-corrected AUC** |
| --- | --- | --- | --- | --- | --- | --- | --- |
| **Predefined severe vs non-severe PTB** | **25** | **30** | **0.8333** | **0.8334** | **0.8371** | **0.7162–0.9310** | **0.8333** |
| **TBscore ≥8 vs <8** | **19** | **36** | **0.8041** | **0.8043** | **0.8079** | **0.6779–0.9067** | **0.8039** |

Supplementary Table S3 ROC analyses were performed using the proportion of KLRG1^⁺^CD28^⁻^CD57^⁺^ cells among Ag85A₄₈-₅₆ tetramer^⁺^CD8^⁺^ T cells as the index marker. The primary analysis used the predefined severe versus non-severe PTB classification applied in the main study. Sensitivity analyses were performed using TB score-defined clinical severity (TB score≥8 vs＜8). Internal stability was assessed by patient-level bootstrap resampling with 10,000 iterations. The apparent AUC represents the AUC estimated directly from the original study cohort. The bootstrap mean AUC and bootstrap median AUC summarize the distribution of AUC estimates across bootstrap samples. The bootstrap 95% confidence interval (CI) represents the corresponding bootstrap-based uncertainty interval. The bootstrap bias-corrected AUC was calculated to account for bootstrap-estimated bias in the apparent in-cohort AUC estimate. These bootstrap analyses assess internal stability and potential bias but do not constitute external validation. Accordingly, all AUC estimates should be regarded as exploratory and hypothesis-generating.
